# Laboratory tests for bovine respiratory bacteria and antimicrobial resistance in commercial feedlot cattle: comparing culture, long-read metagenomics, and recombinase polymerase amplification

**DOI:** 10.3389/fmicb.2026.1806062

**Published:** 2026-05-20

**Authors:** Simon J. G. Otto, Lianne McLeod, E. Luke McCarthy, Tara Funk, Stacey R. Lacoste, Zhijian Chai, Matthew G. Links, Lael D. Barlow, Sheryl P. Gow, Dana Ramsay, Rahat Zaheer, Tim A. McAllister, Paul Stothard, Janet E. Hill, Cheryl L. Waldner

**Affiliations:** 1 HEAT-AMR (Human-Environment-Animal Transdisciplinary AMR) Research Group, School of Public Health, University of Alberta, Edmonton, AB, Canada; 2 Centre for Healthy Communities, School of Public Health, University of Alberta, Edmonton, AB, Canada; 3 Department of Large Animal Clinical Sciences, Western College of Veterinary Medicine, University of Saskatchewan, Saskatoon, SK, Canada; 4 Department of Animal and Poultry Science, College of Agriculture and Bioresources, University of Saskatchewan, Saskatoon, SK, Canada; 5 Department of Veterinary Pathology, Western College of Veterinary Medicine, University of Saskatchewan, Saskatoon, SK, Canada; 6 Department of Agricultural, Food, and Nutritional Science, Faculty of Agricultural, Life, and Environmental Sciences, University of Alberta, Edmonton, AB, Canada; 7 Center for Foodborne, Environmental and Zoonotic Infectious Diseases, Public Health Agency of Canada, Saskatoon, SK, Canada; 8 Lethbridge Research and Development Centre, Agriculture and Agri-Food Canada, Lethbridge, AB, Canada; 9 Department of Veterinary Microbiology, Western College of Veterinary Medicine, University of Saskatchewan, Saskatoon, SK, Canada

**Keywords:** antimicrobial resistance genes, antimicrobial susceptibility testing, bovine respiratory disease, culture, feedlot cattle, long-read metagenomic sequencing, recombinase polymerase amplification

## Abstract

**Introduction:**

The risk to humans and animals from antimicrobial resistance (AMR) has increased the emphasis on antimicrobial stewardship in food animal agriculture. Current stewardship recommendations include increasing diagnostic laboratory testing to inform antimicrobial use for bovine respiratory disease (BRD) management in beef feedlot production, yet the performance of newer molecular and sequencing-based diagnostic tests in commercial settings remains poorly characterized.

**Methods:**

Using nasopharyngeal swabs collected from commercial feedlot calves as part of Canadian surveillance, this study evaluated diagnostic laboratory testing approaches for detecting key bacterial BRD pathogens (*Mannheimia haemolytica, Pasteurella multocida, Histophilus somni*, and *Mycoplasmopsis bovis*) and associated AMR genes. Bayesian latent class models (BLCMs) were applied to compare traditional culture and antimicrobial susceptibility testing (AST) or qPCR with long-read metagenomic sequencing and recombinase polymerase amplification (RPA). Differences in detection of target bacteria and phenotypic or genotypic AMR were assessed across the early feeding period and between age cohorts.

**Results:**

This represents the first large-scale field evaluation of a recently developed, long-read metagenomic sequencing protocol implemented by a commercial laboratory for detecting BRD bacteria and AMR in respiratory samples (*n* = 760) collected by private veterinarians from western Canadian beef feedlots. Detection patterns for BRD bacteria and AMR using culture/AST and metagenomics were often similar between fall-placed calves and yearlings, but with differences from RPA. Detection of BRD bacteria had low sensitivity (< 65% for most organisms/tests), but higher specificity (>90% for all organisms/tests). Detection of macrolide and tetracycline resistance had low but variable sensitivity, with higher estimates for AST compared to metagenomics and RPA, and higher but variable specificity (>90% for most resistance outcomes/tests). Despite not using any targeted enrichment, metagenomic sequencing detected *M. bovis* although with a sensitivity lower than qPCR or RPA. Estimates of predictive value were most informative across the largest range of prevalence for AST, followed by metagenomics and then RPA.

**Discussion:**

This work demonstrates the potential for large scale implementation of long-read metagenomic sequencing to support antimicrobial stewardship and AMR surveillance for feedlot cattle. The estimates of clinical diagnostic performance and predictive values provide evidence-based guidance for three different laboratory tests for BRD management.

## 1 Introduction

The threat of antimicrobial resistance (AMR) to human and animal health has resulted in increasing pressure on food animal agriculture production systems to demonstrate antimicrobial stewardship (McCubbin et al., 2021; Browne et al., 2023; Canadian Academy of Health Sciences, 2025). The World Health Organization Global AMR Action Plan (World Health Organization, 2015), with Canada as a signatory (Public Health Agency of Canada, 2023), identified laboratory diagnostic testing as one of the key strategies to support antimicrobial stewardship in food animal production (World Health Organization, 2018). Currently, most laboratory testing for diagnostics and surveillance of AMR in food animal production continues to rely on traditional bacterial culture and antimicrobial susceptibility testing (AST), which has long turnaround times that contribute to limited use of testing in disease management.

Bovine respiratory disease (BRD) is multifactorial, multi-pathogen, and responsible for the highest morbidity, mortality, and injectable antimicrobial use in North American feedlot cattle (Brault et al., 2019; McAtee et al., 2026). However, laboratory testing has not been commonly adopted for BRD management. A better understanding of the laboratory testing options and performance to identify underlying BRD pathogens and their AMR is critical to protect animal health and welfare and to inform antimicrobial stewardship within the beef industry.

Long-read metagenomic sequencing has been applied successfully to describe BRD bacteria and AMR genes (ARGs) from deep nasopharyngeal swabs (DNPS) from feedlot cattle (Freeman et al., 2022; Herman et al., 2024). Herman et al. (2024) demonstrated the ability to detect important BRD bacteria (*Mannheimia haemolytica, Pasteurella multocida, Histophilus somni, Mycoplasmopsis bovis*, and *Bibersteinia trehalosi*) as well as ARGs within individual reads of BRD bacteria using long-read metagenomic sequencing after a non-specific enrichment protocol. Similar pilot studies of recently developed recombinase polymerase amplification (RPA) assays described the detection of BRD bacteria, ARGs, and integrative conjugative element (ICE)-associated targets with minimal equipment (Conrad et al., 2020; Funk et al., 2025).

Given the importance of BRD and AMR in feedlot production, there is a need to identify laboratory testing strategies that support antimicrobial stewardship for BRD management in Canadian feedlots (Otto et al., 2024; Adewusi et al., 2025). These strategies could support surveillance of BRD bacteria and associated AMR in collaboration with the Canadian feedlot industry (Hannon et al., 2020; CFAASP, 2022, 2025), which currently relies on traditional culture and AST. While initial data have been promising, the application of long-read metagenomic sequencing (Freeman et al., 2022; Herman et al., 2024; Abi Younes et al., 2025) and RPA (Conrad et al., 2020, 2024; Funk et al., 2025, 2026) remain untested on a large scale in commercial feedlots.

The evaluation of new laboratory testing strategies is crucial in food animal production to support testing strategies as part of antimicrobial stewardship. However, there are no gold standards to facilitate the assessment of clinical diagnostic sensitivity and specificity of laboratory tests for BRD bacteria and AMR in feedlot cattle. Bayesian latent class models (BLCM) allow for comparisons of the performance of multiple tests without the requirement that one test is assumed to be a gold standard. This study compared the results of culture or qPCR, AST, long-read metagenomics, and RPA on respiratory samples for detection of BRD bacteria and AMR. Samples were collected from commercial feedlot cattle by private veterinary clinics and were shipped to regional commercial and research laboratories. For the purposes of the comparisons in this study, AMR was defined as detection of phenotypic AMR by culture and AST or detection of ARGs by metagenomics or RPA.

The specific objectives of this study were to: (1) collaborate with Canadian feedlot surveillance to deploy a protocol for long-read metagenomic sequencing and RPA testing on DNPS collected from cattle in commercial western Canadian feedlots; (2) use BLCMs to compare the test performance of traditional culture and AST or qPCR to both long-read metagenomic sequencing and RPA for detection of BRD bacteria of interest (*M. haemolytica, P. multocida, H. somni*, and *M. bovis*), AMR of interest (macrolide, tetracycline, and phenicol resistance), and ICE-associated targets; and (3) compare the risk for detection of BRD bacteria and AMR between animal type—fall-placed calves (FPC) and yearling cattle (YRL)—and sampling time—near arrival and 2–3 weeks into the feeding period.

## 2 Materials and methods

### 2.1 Ethics statement

This study was conducted in accordance with the recommendations of the Canadian Council of Animal Care (CCAC; Olfert et al., 1993). An ethics protocol and standard operating procedure for deep nasopharyngeal swab collection were developed and approved by the Animal Care and Use Committee at the University of Alberta (ACUC Livestock—University of Alberta AUP00004110) and shared with the Research Ethics Board at the University of Saskatchewan (USask AREB File Number 20220072) and the Animal Care Committee of Feedlot Health Management Services as a study partner.

### 2.2 Study population and sampling procedure

The present study was carried out in collaboration with the Canadian Feedlot Antimicrobial Use and Antimicrobial Resistance Surveillance Program (CFAASP) of the Canadian Integrated Program for Antimicrobial Resistance Surveillance (CIPARS) as part of a nationwide, longitudinal survey of AMR in feedlot cattle (Hannon et al., 2020; CFAASP, 2022, 2025). The CFAASP program partnered with consulting veterinary practices that identified volunteer feedlots intended to represent the distribution of feedlot sizes and geographic locations within the western Canadian feedlot industry. As part of the annual DNPS collection for culture and susceptibility testing, an additional DNPS was collected for metagenomic sequencing and RPA testing from a subset of CFAASP participants.

Registered veterinary technologists from three private veterinary clinics sampled cattle from 19 commercial feedlots in Alberta between September 2022 and January 2023 with testing intended to coincide with peak arrival of recently weaned beef calves. The veterinary clinics submitted samples to a commercial diagnostic laboratory (Prairie Diagnostic Services (PDS), Saskatoon, SK). The identities of the feedlots were known only by the clinics to maintain confidentiality. Cattle were restrained in commercial cattle chutes and headgates equipped with neck extenders for the collection of swabs. For this study, two DNPS were collected from a convenience sample of 20 cattle from 13 pens of FPC and 6 pens of YRL at arrival processing and before metaphylaxis. Another convenience sample of 20 cattle was sampled from the same pens at approximately 14 days on feed (DOF); the same cattle were not targeted for resampling within each pen.

The external nares were wiped with a single-use paper towel and a double-guarded DNPS (Continental Plastic Corp., Delevan, WI, USA) was directed into the ventral meatus of the nostril. The polyester-tipped swab was advanced through the inner sheath and vigorously rotated against the nasopharyngeal mucosa for 5–6 rotations. The swab was withdrawn into the inner sheath and outer guard prior to removal from the nostril. The second sample was obtained from the alternate nostril. The swabs were each placed into 1 mL Amies transport media (Micronostyx, Ottawa, Canada), the excess lengths cut off, and the tubes sealed. Samples were couriered to the laboratory in an insulated cooler with ice packs.

Metadata available from the CIPARS submission sheet included the age group (fall-placed calf/yearling), average weight of cattle in the pen at arrival, and DOF for the second sampling time point. Due to the anonymity of the feedlots, the research team had no access to additional metadata such as farm of origin, BRD incidence, history of metaphylaxis, or other antimicrobial treatments.

### 2.3 Methods for bacterial culture and antimicrobial susceptibility testing

Methods for culture, identification, and AST for *M. haemolytica, P. multocida*, and *H. somni* have been previously described (Abi Younes et al., 2024). Briefly, plates were inoculated with the DNPS tip from one swab on the same day the samples arrived at the laboratory. The swab tip was then stored at −80 °C for subsequent DNA extraction for *M. bovis* qPCR and RPA analysis. Bacterial species from culture plates were identified using matrix-assisted laser desorption/ionization time-of-flight mass spectrometry (MALDI-TOF MS, Bruker Daltonik, Bremen, Germany) and Biotyper Microflex LT Compass version 1.4 software (Bruker Corporation, Billerica, MA) according to manufacturer guidelines.

For AST, isolates were streaked onto purity plates specific for *M. haemolytica, P. multocida*, and *H. somni*. One colony of interest from each purity plate was selected for AST completed using a commercially available bovine serial broth microdilution panel (Thermo Fisher Scientific™, Waltham, MA, USA, Bovine AST BOPO7F Plate) on the Sensititre™ platform. The minimum inhibitory concentrations (MICs) were read on the BIOMIC^®^ V3 microplate reader (Giles Scientific Inc., Santa Barbara, CA). The MIC for each antimicrobial was compared to Clinical and Laboratory Standards Institute (CLSI) breakpoints, where available (CLSI, 2024). For this analysis, there were CLSI breakpoints for tulathromycin, gamithromycin, tildipirosin, tetracycline, and florfenicol for *M. haemolytica, P. multocida* and *H. somni*; for tilmicosin, a breakpoint was only available for *M. haemolytica*; results were not reported where breakpoints did not exist.

### 2.4 Non-specific enrichment and extraction for metagenomic sequencing

The second set of DNPS was stored at 4 °C for no more than 8 h after arrival until processing. Non-specific bacterial enrichment as previously described (Abi Younes et al., 2025) was performed with the only difference being that the enrichment time was 14 h compared to the previous study that used 14 h in 2020 and 10 h in 2021. Samples were frozen immediately after enrichment at −80 °C for a maximum of 5 months before DNA extraction. For DNA extraction, the only difference was that an aliquot (4.0 mL) of the enriched media was pelleted, resuspended, treated with DNase (Invitrogen, Waltham, MA, USA), inactivated via heat treatment and washed. DNA extraction was performed via alcohol precipitation, using the Qiagen Puregene Buccal Cell Kit (QIAGEN Inc., Germantown, MD, USA) as previously described (Abi Younes et al., 2025). Extracted DNA was stored at 4 °C until sequencing. A minimum of 2 μg DNA (50 ng/μL in 40 μL) was required for library preparation. Samples were tested using Qubit 1x dsDNA HS Assay kit (Invitrogen, Waltham, MA, USA) and 211 of the samples were re-extracted from the reserve enriched aliquots to obtain additional DNA.

### 2.5 Library preparation and sequencing for bacterial metagenomic sequencing

Extracted DNA from 760 samples was purified and size selected with 0.4x AMPure XP beads (Beckman Coulter, Indianapolis, IN, USA). Sample DNA was size selected and normalized to 400 ng total prior to library preparation, where sufficient DNA was present. For 215 samples with insufficient DNA for size selection; non-size selected DNA was used.

Library preparation was completed with a Hamilton NGS STAR automation workstation, following a modified in-house protocol for automation based on Oxford Nanopore Technologies (ONT) ligation protocol with native barcoding (SQK-LSK109 and EXP-NBD196, ONT, Oxford, UK) in a 96-well plate high-throughput library format with minor modifications to minimize barcode crossover. Barcode ligation was followed by the addition of 1 μL of EDTA (Invitrogen, Waltham, MA, USA), a 10 min room temperature incubation and a 10 min 65 °C incubation. Barcoded DNA samples for a single pen from timepoints one and two were created in libraries of eight samples that were then pooled together into libraries of 40 samples along with library preparation negative controls (nuclease free water, Invitrogen, Waltham, MA, USA), for a total of 19 libraries. Extraction controls for each set of samples were processed as a separate library.

Fifty ng of each prepared library was sequenced at the Omics and Precision Agriculture Laboratory (OPAL, University of Saskatchewan, Saskatoon, SK, CA) on the PromethION24 platform. Pooled libraries were loaded onto flow cells (FLO-PRO114M; ONT version R10.4.1, Oxford Nanopore Technologies, Oxford, UK). Sequencing was conducted over 48 h using default run parameters and base-calling using a high accuracy model with a Q-score cut off of nine.

### 2.6 Bioinformatic processing for bacterial metagenomic sequencing

Data from the MinKnow workflow were processed with Porechop v0.2.4 (Wick et al., 2017) for quality control and Nanofilt v2.8.0 (De Coster et al., 2018) to remove adapters and short (< 200 bp) reads. NanoStat v1.6.0 (De Coster et al., 2018) provided statistics on filtered data.

Taxonomic classification of reads was done with Kraken 2 v2.1.2 (Wood et al., 2019), using a custom database that included bacterial, viral, and archaeal subsets of the November 2023 RefSeq database (Goldfarb et al., 2025) as well as the *Bos taurus* ARS-UCD1.2_Btau5.0.1Y genome assembly, which consists of the ARS-UCD1.2 genome assembly (Rosen et al., 2020) and the Y chromosome sequence from Btau5.0.1 (Elsik et al., 2009) as previously described in detail (Abi Younes et al., 2025). Host-filtered reads (i.e., those not assigned to *B. taurus*) were extracted using the KrakenTools v1.2 (Lu et al., 2022) utility extract_kraken_reads.py. Bracken version 2.7 (Lu et al., 2017) with a minimum read length of 200 base pairs (“–read-length 200”) was used to improve the species-level estimation of abundance. Reads classified as host were removed from further consideration. A custom script, report_taxon_read_lengths.py, added the total amount of sequence in base pairs reported for each species (including child taxa) and the fraction of total classified sequence.

Non-host reads were converted from FASTQ to FASTA format using Seqtk v1.3 (Li, 2018), then used to search for ARGs. Antimicrobial resistance genes were identified in non-host reads using Abricate (version 1.0.0; Seemann, 2020) and AMRFinderPlus (version 3.11.18; Feldgarden et al., 2019, 2021), both with the NCBI Bacterial Antimicrobial Resistance Reference Gene Database (version 2023-11-15.1). Abricate was also run using the Comprehensive Antimicrobial Resistance Database (CARD; version 3.2.8; Alcock et al., 2023; https://card.mcmaster.ca/download). For AMRFinderPlus, the minimum percent identity and percent coverage thresholds were set to 80% and the -plus option. Default parameters were used for Abricate (80% minimum percent identity and percent coverage). Results generated by ARG searching with NCBI and CARD databases were merged based on gene name and start/stop coordinates. Once merged, the CARD gene names were preferentially used in downstream reports.

Two custom sequences were selected for detection of ICE-associated targets from BRD organisms (Conrad et al., 2024), which included regions from pairs of adjacent genes: the *tet(H)* gene and either *tnpA* [*tnpA-tet(H)*] or *ebrB* [*ebrB-tet(H)*]. These were generated using *in silico* PCR (Ozer, 2022) to extract the sequences as amplicons from *P. multocida* assembly ASM2987329v1 (GCF_029873295.1) and *H. somni* assembly ASM216229v1 (GCF_002162295.1). A custom script (parse_amplicon_blast_results.py) ensured that the amplicon lengths were within one base pair of the expected length. These amplicon sequences were then used as BLAST queries [BLAST+ v2.12.0 (Camacho et al., 2009)] to search a subset of reads identified as *M. haemolytica, P. multocida*, or *H. somni* at 90% identity and 80% coverage of the query sequence.

Serotype-specific genomic regions identified in *M. haemolytica* (Klima et al., 2017) were used as BLAST queries [BLAST+ v2.12.0 (Camacho et al., 2009)] to search reads classified as *M. haemolytica* by Kraken. A custom script (parse_serotyping_results.py) was used to filter results for a maximum E-value of 1E-30 and minimum alignment length of 100 base pairs and verify that no read was retrieved as a BLAST hit by two different serotype sequences.

### 2.7 DNA extraction for RPA and qPCR

Following collection of a sample for bacterial culture, the remaining volume from the raw sample was used for DNA extraction, which was completed using the MagMAX™ CORE Nucleic Acid Purification Kit (Thermo Fisher Scientific) according to manufacturer guidelines, and the KingFisher™ Flex Purification System with a 96 PCR head (Thermo Fisher Scientific). The process yielded an approximate final elution volume of 100 μL of extracted DNA from each sample, which was stored at 4 °C until RPA analysis and testing of *M. bovis* using qPCR.

### 2.8 RPA testing

Extracted DNA was tested using published RPA assays for BRD-associated bacteria (*M. haemolytica* [serotypes A1 and A6 only], *P. multocida, H. somni*, and *M. bovis*; Conrad et al., 2020), ICE-associated target variants (Conrad et al., 2024), and macrolide ARGs (Funk et al., 2025; Table 1). Only samples that were RPA positive for *M. haemolytica, P. multocida*, or *H. somni* were tested again for ICE-associated target variants [*tnpA-tet(H), ebrB-tet(H)*] and macrolide resistance genes [*msrE*-*mphE, erm(42)*].

**Table 1:** Gene target list for the detection of bovine respiratory disease (BRD) bacteria, integrative and conjugative elements, and macrolide antimicrobial resistance genes using recombinase polymerase amplification (RPA).

Target	Gene	Assay type	References
BRD bacteria			
*Mannheimia haemolytica* (serotypes A1, A6)	*nma*A	Multiplex	Conrad et al., 2020
*Pasteurella multocida*	*kmt*1	Multiplex	Conrad et al., 2020
*Histophilus somni*	Hs_0116	Multiplex	Conrad et al., 2020
*Mycoplasmopsis bovis*	*uvrC*	Multiplex	Conrad et al., 2020
Antimicrobial resistance determinants			
Macrolide ARG	*msrE, mphE*	Multiplex	Funk et al., 2025
Macrolide ARG	*erm(42)*	Multiplex	Funk et al., 2025
ICE-associated target variant ICEtnpA [*tnpA-tet(H)*]	*tet(H)/tnpA*	Multiplex	Conrad et al., 2020
ICE-associated target variant ICEebrB [*ebrB-tet(H)*]	*tet(H)/ebrB*	Multiplex	Conrad et al., 2024

ARG, antimicrobial resistance gene; ICE, integrative conjugative element.

Specific bacterial presence was assessed using real-time RPA for BRD bacteria and ICE-associated target variants (Table 1). Reactions were performed at 37 °C for 33 min on a T16-ISO machine (Axxin Ltd, Fairfield, Victoria, AUS), using a 50 μL reaction volume in 0.2 mL strip tubes. A fluorescence threshold of ≥100 mV for at least 60 s was used to define a “positive” fluorescence signal for all reactions (Conrad et al., 2020, 2024).

Testing for macrolide ARGs was completed similarly but at 39 °C for 26 min. Classification of RPA-positive samples employed an amended fluorescence threshold algorithm of ≥400 mV for at least 60 s for macrolide ARGs to account for non-selective background fluorescence observed during assay development (Funk et al., 2025).

### 2.9 *M. bovis* qPCR testing

qPCR testing was performed for *M. bovis* rather than culture. *M. bovis uvrC* target gene copies were quantified on extracted DNA by qPCR on a BioRad CFX96 Real Time PCR Detection System (BIO-RAD, Hercules, CA, USA) as previously described (Donbraye et al., 2026a). Enzyme activation was completed for 2 min at 95 °C, followed by 40 cycles of amplification, consisting of 5 s at 95 °C and 33 s at 60 °C. Samples were considered “negative” if no amplification was detected, “suspect” for *M. bovis* at a cycle threshold (Ct) value of ≥37, and “positive” for the presence of *M. bovis* at a Ct value of < 37.

### 2.10 Data management and descriptive analyses

Animal data, culture and AST, summary taxonomy, and ARG results from metagenomic sequencing, RPA data, and qPCR were managed in a spreadsheet (Microsoft Excel, version 2401, Microsoft Corporation, Redmond, Washington, DC, USA), and descriptive analyses were completed using a commercial statistical software package (Stata/BE, version 19.0, StataCorp LLC, College Station, TX, USA).

Culture results and results of AST for the antimicrobials considered in the Bayesian latent class models (BLCMs) were summarized for *M. haemolytica, P. multocida*, and *H. somni* isolates. For the purposes of the primary BLCM analysis, isolates were considered susceptible if the AST results were reported as susceptible or intermediate. The median total read length in base pairs (bp), median read length (bp), theoretical coverage, and number of reads detected by long-read metagenomic sequencing for *M. haemolytica, P. multocida, H. somni*, and *M. bovis* were summarized for all samples. Theoretical coverage was calculated by dividing the total read length for each bacterium in megabase pairs by the size of its reference genome (*M. haemolytica*: 2.8 Mb [NCBI GCF_002285575.1], *P. multocida*: 2.3 Mb [NCBI GCF_002073255.2], *H. somni*: 2.3 Mb [NCBI GCF_000019405.1], and *M. bovis*: 0.9 Mb [NCBI GCF_001930225.1]). Specific resistance genes for macrolides, tetracyclines, and phenicols were identified based on their clinical importance for BRD management. The frequency with which any of *msrE* and/or *mphE, erm(42), estT, tet(H), floR*, or at least one of the ICE-associated target variant reads were detected within *M. haemolytica, P. multocida*, or *H. somni* reads were also summarized. The frequency of samples identified with *M. haemolytica* classified as either serotype A1 or A6 by long-read metagenomic sequencing was also reported to facilitate direct comparison to the RPA assay for *M. haemolytica* that also only detects these serotypes. For RPA, the frequency of detection of *M. haemolytica, P. multocida, H. somni*, and *M. bovis* was reported, along with frequency with which *msrE-mphE, erm(42)*, or the ICE-associated target variants *tnpA-tet(H)* or *ebrB-tet(H)* were detected. If the sample was RPA negative for *M. haemolytica, P. multocida*, and *H. somni*, it was not tested further and considered negative for ICE-associated target variants and ARGs.

### 2.11 Bayesian latent class models

Bayesian latent class models were developed to estimate the diagnostic sensitivity and specificity of each of the three tests (culture ± AST for *M. haemolytica, P. multocida, H. somni* or qPCR for *M. bovis*, metagenomics, and RPA) for (1) detection of *M. haemolytica, P. multocida, H. somni*, and *M. bovis*, and (2) detection of samples with isolates of *M. haemolytica, P. multocida*, or *H. somni* with resistance to specific antimicrobials considered important in BRD-associated bacteria. Detection of AMR in any of *M. haemolytica, P. multocida*, and *H. somni* was assessed by integrating the per sample phenotypic AST results coincident with metagenomic- or RPA-based detection of ARGs corresponding to specific drug classes. Targets for modeling were identified based on prevalence of resistance and ARGs as well as potential clinical importance of the antimicrobial drug and AMR to BRD management, including macrolides, tetracyclines, and florfenicol. Antimicrobials for which CLSI cutoffs were not available for at least one organism were not considered.

**Figure 1:**
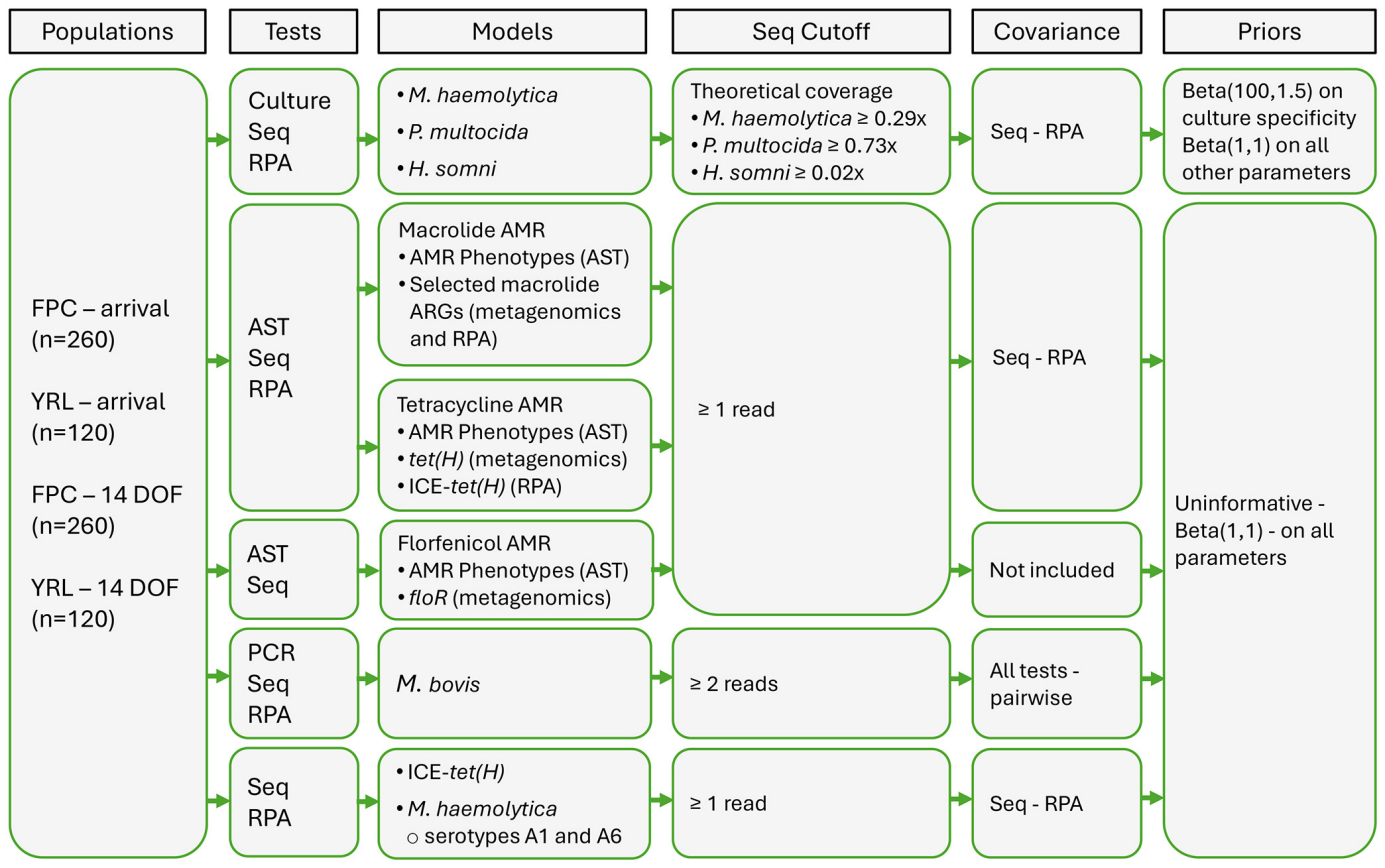
Summary of key components for Bayesian latent class models. Models compared combinations of tests and outcomes from deep nasopharyngeal swabs collected across four populations of feedlot cattle. FPC, fall-placed calves; YRL, yearlings; DOF, days on feed; Seq, long-read metagenomic sequencing; RPA, recombinase polymerase amplification; AST, antimicrobial susceptibility testing; PCR, polymerase chain reaction; AMR, antimicrobial resistance; ICE-*tet(h)*, integrative and conjugative elements, ICE-associated target variants *tnpA-tet(H), ebrB-tet(H)*.

Three-test (long-read metagenomic sequencing vs. RPA vs. culture/AST or qPCR), four-population BLCMs were primarily used for the analysis. The four populations included FPC sampled at arrival, YRL sampled at arrival, FPC sampled at 14 DOF, and YRL sampled at 14 DOF. All models included covariance terms between the sensitivities and specificities of metagenomics and RPA, recognizing that both tests were DNA-based and that the results could not be assumed to be independent. Additionally, in *M. bovis* models that included qPCR, covariance terms between qPCR and metagenomics and qPCR and RPA were also included. Key components of the full set of BLCMs are summarized in Figure 1. Development of thresholds for the classification of metagenomic sequencing results for bacteria as positive or negative were described in detail in Supplementary materials 1, along with detailed descriptions of the models and corresponding sensitivity analyses.

Models were run using JAGS software v4.3.0 (Plummer, 2003) and the runjags package (Denwood, 2016) in R v4.1.0 (R Core Team, 2021). Uninformative priors (beta[1,1]) were used for estimated parameters, except for models for detection of *M. haemolytica, P. multocida*, and *H. somni*, in which a prior [beta(100,1.5)] was incorporated for culture specificity. Visual inspection of trace, distribution, and autocorrelation plots along with diagnostics including potential scale reduction factor (< 1.05), effective sample size (>1,000), and Monte Carlo standard errors as a percent of standard deviation (< 5%) were used to evaluate model convergence and deemed satisfactory for all models. Results were reported as medians of the posterior distributions along with 95% credible intervals (CrIs). Results were considered significantly different if the CrIs did not overlap. Estimates of positive and negative predictive value (PPV, NPV) were generated for key test combinations across a range of possible pre-test probabilities for detection of respiratory bacteria and macrolide or tetracycline AMR (Sergeant, 2018).

### 2.12 Differences in detection of bacteria, ARGs, and ICE-associated target variants among time points

Generalized estimating equations (GEE) were used for each set of test results to describe the prevalence and variability of bacterial species of interest and AMR/ARG detection between samples at arrival and samples collected at 14 DOF for the FPC and YRL and between FPC and YRL at each collection time (STATA 19.0, StataCorp, College Station, TX). Differences were analyzed using models with a logit link function, binomial distribution, and robust variance accounting for clustering by pen/feedlot. Fixed effects included sampling time and animal age group as well as an interaction term between sampling time and animal age group; time between sample collection and arrival at the laboratory (transit/shipment time in days) was also included as a fixed effect to adjust for any impact of delays and resulting decreased sample quality. When GEE models failed to converge due to sampling time points with few or zero positive samples, exact logistic regression models were used to generate estimates. Results were reported as odds ratios (OR) with 95 percent confidence intervals (95% CI). *P*-values < 0.05 were considered statistically significant.

## 3 Results

Deep nasopharyngeal swabs were collected from 20 cattle each of 19 pens from 19 anonymous, geographically distributed feedlots in western Canada at the two time points (*n* = 760 total). Of these, there were 520 samples from FPC in 13 pens and 240 YRL in six pens, each divided equally across the two time points (arrival *n* = 380; 14 DOF *n* = 380). Samples were collected by registered veterinary technologists from three participating feedlot veterinary clinics located approximately 600 km (±20 km) from the receiving laboratory. Initial sampling took place at arrival processing, with most of the second samples collected near the intended target of 14 DOF (median, 14 DOF; range 10–23 DOF). Greater than 97% of the samples arrived at the laboratory within 5 days of collection with 82% arriving within 2 days. The average reported weight at arrival processing for the FPC was 266 kg (standard deviation, SD 43 kg), while for the YRL the mean weight was 399 kg (SD 66 kg).

All samples (*n* = 760) were tested using culture, long-read metagenomic sequencing, and RPA for the detection of *M. haemolytica, P. multocida*, and *H. somni*, as well as detection of *M. bovis* via qPCR. Tetracycline and florfenicol AMR results were available for all samples and tests. Similarly, macrolide AMR results were available for all samples and tests except for two samples where there was insufficient sample volume for macrolide ARG RPA testing.

Across age groups and sampling times, culture detected *M. haemolytica* in 183/760 (24%) samples, *P. multocida* in 177/760 (23%) samples, and *H. somni* in 42/760 (5.5%) samples (Table 2). Detection frequencies varied by bacterial species, age group, and sampling time (Table 2). The number of samples yielding isolates resistant to target macrolides or tetracycline similarly varied, primarily by age group and sampling time (Table 2). No florfenicol resistant isolates were recovered from FPC or YRL samples at arrival. At 14 DOF, florfenicol resistance was detected in < 2% of FPC- and < 1% of YRL-derived isolates (Table 2). Complete culture and AST results were summarized at both the isolate and sample levels by CFAASP (Supplementary materials 2: Figures S2.1–S2.14).

**Table 2:** Number (%) of samples culture-positive for *M. haemolytica, P. multocida*, and *H. somni*, along with number (%) of samples in which any of *M. haemolytica, P. multocida*, or *H. somni* were classified as resistant by antimicrobial susceptibility testing (AST^a^) for fall-placed calves (FPC) and yearlings (YRL) from commercial feedlots (*n* = 760 deep nasopharyngeal swab samples, *N* = 19 feedlots).

Test	FPC		YRL	
	Arrival	14 DOF	Arrival	14 DOF
**Culture**	*n =* 260	*n =* 260	*n =* 120	*n =* 120
*M. haemolytica*	56 (22%)	65 (25%)	26 (22%)	36 (30%)
*P. multocida*	92 (35%)	44 (17%)	24 (20%)	17 (14%)
*H. somni*	15 (5.8%)	25 (9.6%)	1 (0.8%)	1 (0.8%)
**AST** ^**a**^ **–Macrolides**	*n =* 260	*n =* 258^b^	*n =* 120	*n =* 120
Resistant to GAM, TULA, TILD or TILM^c^	7 (2.7%)	88 (34%)	6 (5.0%)	13 (11%)
Resistant to GAM or TULA	7 (2.7%)	83 (32%)	6 (5.0%)	13 (11%)
Resistant to TILD or TILM^c^	6 (2.3%)	75 (29%)	5 (4.2%)	13 (11%)
**AST** ^**a**^ **–Tetracycline**	*n =* 260	*n =* 260	*n =* 120	*n =* 120
Resistant to tetracycline	2 (0.8%)	55 (21%)	4 (3.3%)	11 (9.2%)
**AST** ^**a**^ **–Phenicols**	*n =* 260	*n =* 260	*n =* 120	*n =* 120
Resistant to florfenicol	0 (0.0%)	5 (1.9%)	0 (0.0%)	1 (0.8%)

DOF, days on feed; GAM, gamithromycin; TULA, tulathromycin; TILD, tildipirosin; TILM, tilmicosin.

^a^For AST, intermediate MICs were classified as susceptible.

^b^Macrolide results were not available for two recombinase polymerase amplification samples due to insufficient volume for testing.

^c^TILM results were limited to *M. haemolytica* as there were no CLSI breakpoints for *P. multocida* and *H. somni* (CLSI VET01S Ed7).

After demultiplexing, trimming, and quality filtering nanopore data, 715.4 million long-read metagenomic reads were obtained. Following the removal of host-related sequences, which made up 18.0% (128.6 million reads) of total reads, 586.8 million non-host reads remained. Of these, 578.2 million reads were classified as bacterial, accounting for 80.8% of the total reads. Another 3.3 million reads (0.46% of total reads) were classified as viral and 6,491 reads (0.001%) were classified as archaeal, while 5.3 million reads (0.74% of total reads) remained unclassified or had ambiguous classification.

The median total base pairs per sample, median theoretical coverage, and median number of reads along with the number of samples with ≥1 read were greater for *M. haemolytica* and *P. multocida* than for *H. somni* (Table 3). The median per sample read lengths were more comparable for *M. haemolytica, P. multocida*, and *H. somni*. All sequencing metrics were substantially lower for *M. bovis*.

**Table 3:** Summary of sequence statistics for *M. haemolytica, P. multocida, H. somni*, and *M. bovis* in fall-placed calves and yearlings from commercial feedlots (*n* = 760 deep nasopharyngeal swab samples, *N* = 19 feedlots).

Bacteria	Number (%) of samples with ≥1 read	Median total base pairs	Median per sample read length	Median theoretical coverage ( × )	Median number of reads
*M. haemolytica*	705 (93%)	101,512	1,421	0.036	46
*P. multocida*	721 (95%)	84,485	1,477	0.037	35
*H. somni*	598 (79%)	7,786	1,570	0.003	4
*M. bovis*	156 (21%)	2,582	936	0.003	1

Samples which have at least 1 read classified as the corresponding species are reported. Median theoretical coverage is calculated for each species based on the respective genome size. Reference genomes: *M. haemolytica*: 2.8 Mb [NCBI GCF_002285575.1]; *P. multocida*: 2.3 Mb [NCBI GCF_002073255.2]; *H. somni*: 2.3 Mb [NCBI GCF_000019405.1]; and *M. bovis*: 0.9 Mb [NCBI GCF_001930225.1].

Detection of ARGs were linked with the bacterial species classification at the individual read level. The most prevalent and potentially clinically relevant ARGs identified in reads classified as *M. haemolytica, P. multocida*, or *H. somni* were *tet(H), floR, estT, mphE*, and *msrE* (Table 4); all other ARGs identified in ≤ 25 samples are not reported. While *erm(42)* was identified in only 18 samples, it was included in the BLCMs as it was a target of the multiplex macrolide RPA assay.

**Table 4:** Number (%) of samples in which antimicrobial resistance genes (ARGs) were detected by long-read metagenomics in reads classified as *M. haemolytica, P. multocida*, or *H. somni* from fall-placed calves and yearlings from commercial feedlots (*n* = 760 deep nasopharyngeal swab samples, *N* = 19 feedlots).

Gene	Resistance class	Number (%) of samples with ≥1 read of an ARG associated with target bacteria
*tet(H)*	Tetracyclines	99 (13.0%)
*sul2*	Sulfonamides	90 (11.8%)
*floR*	Phenicols	76 (10.0%)
*aph(3″)-Ib*	Aminoglycosides	75 (9.9%)
*aph(6)-Id*	Aminoglycosides	71 (9.3%)
*aph(3′)-Ia*	Aminoglycosides	68 (8.9%)
*tet(A)*	Tetracyclines	34 (4.5%)
*tet(B)*	Tetracyclines	34 (4.5%)
*estT*	Macrolides	26 (3.4%)
*mphE*	Macrolides	26 (3.4%)
*msrE*	Macrolides	26 (3.4%)

Displayed ARGs were identified in more than 25 samples.

Detection of reads containing *msrE-mphE, estT, erm(42), tet(H)*, and *floR*, as well as any genes associated with macrolide or tetracycline resistance, varied by age group and sampling time (Table 5). The percentage of samples in which long-read metagenomic sequencing detected ICE-associated target variants *tnpA-tet(H)* and *ebrB-tet(H)*, as well as the percentage of samples with detected *M. haemolytica* genes associated with serotype-specific genomic regions, also varied by age group and sampling time (Table 5).

**Table 5:** Summary of results of long-read metagenomic sequencing for the detection of selected antimicrobial resistance genes (ARGs), integrative conjugative element (ICE)-associated target variants, and genes associated with *M. haemolytica* serotypes.

Test	FPC		YRL	
	Arrival	14 DOF	Arrival	14 DOF
**Macrolide ARGs**	*n =* 260	*n =* 258^a^	*n =* 120	*n =* 120
*mphE*−*msrE*^b^	0 (0%)	18 (7.0%)	2 (1.7%)	7 (5.8%)
*estT*	7 (2.7%)	18 (7.0%)	0 (0%)	1 (0.8%)
*erm(42)*	0 (0%)	17 (6.6%)	0 (0%)	0 (0%)
*msrE*−*mphE*^b^ or *erm(42)*	0 (0%)	29 (11%)	2 (1.7%)	7 (5.8%)
*msrE*−*mphE*^b^*, erm(42)* or *estT*	7 (2.7%)	37 (14%)	2 (1.7%)	8 (6.7%)
Any macrolide-associated ARG^c^	29 (11%)	49 (19%)	9 (7.5%)	14 (12%)
**Tetracycline ARGs**	*n =* 260	*n =* 260	*n =* 120	*n =* 120
*tet(H)*	27 (10%)	62 (24%)	2 (1.7%)	8 (6.7%)
Any tetracycline-associated ARG^d^	57 (22%)	88 (34%)	11 (9.2%)	24 (20%)
**Phenicol ARGs**	*n =* 260	*n =* 260	*n =* 120	*n =* 120
*floR*	10 (3.8%)	44 (17%)	15 (13%)	7 (5.8%)
**ICE-associated target variants**	*n =* 260	*n =* 260	*n =* 120	*n =* 120
*tnpA-tet(H)*	17 (6.5%)	53 (20%)	0 (0%)	5 (4.2%)
*ebrB-tet(H)*	11 (4.2%)	15 (5.8%)	1 (0.8%)	3 (2.5%)
*tnpA-tet(H)* or *ebrB-tet(H)*	27 (10%)	64 (25%)	1 (0.8%)	7 (5.8%)
***M. haemolytica*** **serotypes**	*n =* 260	*n =* 260	*n =* 120	*n =* 120
Serotype A1	19 (7.3%)	17 (6.5%)	2 (1.7%)	3 (2.5%)
Serotype A2	101 (39%)	79 (30%)	51 (43%)	57 (48%)
Serotype A6	20 (7.7%)	2 (0.8%)	3 (2.5%)	12 (10%)
Serotype A1 or A6	37^e^ (14%)	19 (7.3%)	5 (4.2%)	15 (13%)

Counts represent the number of samples in which the targeted genes were detected on at least one read identified as *M. haemolytica, P. multocida*, or *H. somni* from samples used in the Bayesian latent class models for fall-placed calves (FPC) and yearlings (YRL) from commercial feedlots (*n* = 760 deep nasopharyngeal swab samples, *N* = 19 feedlots). DOF, days on feed.

^a^Macrolide results were not available for two RPA samples due to insufficient volume for testing.

^b^*msrE-mphE*: includes any samples positive for *msrE* or *mphE*.

^c^Other macrolide-associated ARGs included: *crp, ermB, ermC, ermD, ermT, Escherichia coli emrE, evgA, evgS, gadW, gadX, hns, Klebsiella pneumoniae kpnH, mdtE, mdtF, mef(F), mphK, msr(G), tolC*.

^d^Other tetracycline-associated ARGs included: *acrB, AcrS, emrK, emrY, Enterobacter cloacae acrA, Escherichia coli acrA, Escherichia coli mdfA, evgA, evgS, hns, marA, mgrA, oqxA, oqxB, tet(39), tet(A), tet(B), tet(C), tet(K), tet(L), tet(M), tet(X3), tolC, ykkC, ykkD*.

^e^Both Serotype A1 and A6 were detected in 2 samples.

Across age groups and sample times, RPA detected *M. haemolytica* (limited to serotypes A1 and A6) in 91/760 (12%) samples, *P. multocida* in 86/760 (11%) samples, *H. somni* in 63/760 (8.3%) samples, and *M. bovis* in 153/760 (20%) samples (Table 6). The number of samples in which RPA detected these bacteria, along with one or both ICE-associated target variants, *msrE-mphE*, or *erm(42)*, were summarized by age group and time point (Table 6).

**Table 6:** Summary of results for detection of *M. haemolytica, P. multocida, H. somni, M. bovis*, ICE-associated target variants, and *msrE-mphE* or *erm(42)* by recombinase polymerase amplification (RPA) from samples considered in Bayesian latent class models for fall-placed calves (FPC) and yearlings (YRL) from commercial feedlots (*n* = 760 deep nasopharyngeal swab samples, *N* = 19 feedlots).

Test	FPC		YRL	
	Arrival	14 DOF	Arrival	14 DOF
**Bacteria**	*n =* 260	*n =* 260	*n =* 120	*n =* 120
*M*.*haemolytica*^a^ (Serotypes A1 and A6)	42 (16%)	26 (10%)	5 (4.2%)	18 (15%)
*P. multocida*	51 (20%)	12 (4.6%)	13 (11%)	10 (8.3%)
*H. somni*	27 (10%)	23 (8.8%)	6 (5.0%)	7 (5.8%)
*M. bovis*	26 (10%)	83 (32%)	16 (13%)	28 (23%)
**ICE-associated target variants**	*n =* 260	*n =* 260	*n =* 120	*n =* 120
*tnpA-tet(H)*	13 (5.0%)	22 (8.5%)	3 (2.5%)	8 (6.7%)
*ebrB-tet(H)*	17 (6.5%)	7 (2.7%)	6 (5.0%)	13 (11%)
*tnpA-tet(H)* or *ebrB-tet(H)*	29 (11%)	26 (10%)	9 (7.5%)	18 (15%)
**ARG**	*n =* 260	*n* = 258^b^	*n =* 120	*n =* 120
*msrE-mphE*	80 (31%)	35 (14%)	16 (13%)	27 (23%)
*erm(42)*	30 (12%)	20 (7.8%)	8 (6.7%)	8 (6.7%)
*msrE-mphE* or *erm(42)*	80 (31%)	38 (15%)	17 (14%)	27 (23%)

ICE, integrative and conjugative element; ARG, antimicrobial resistance gene.

^a^The RPA assay detects serotypes A1 and A6.

^b^Macrolide results were not available for two RPA samples due to insufficient volume for testing.

A small percentage of FPC samples were positive by qPCR for *M. bovis* at arrival (9/260, 3.5%), while 117/260 (45%) samples were positive at 14 DOF. Among YRL, 15/120 (13%) samples were positive for *M. bovis* by qPCR on arrival, while 33/120 (28%) samples were positive at 14 DOF.

### 3.1 Bayesian latent class models: *M. haemolytica, P. multocida*, and *H. somni*

The process of setting the final theoretical coverage cutoffs for determining positivity of samples for *M. haemolytica, P. multocida*, and *H. somni* is described in Supplementary materials 1. The final theoretical coverage cutoff for *M. haemolytica* determined by BLCM comparison to culture and RPA was 0.29X, a 2.5-fold increase from the ROC-suggested cutoff (0.12X) to meet the ≥0.90 threshold for metagenomic specificity (Supplementary materials 3: Table S3.1). Similarly, the ROC-determined cut off for *P. multocida* of 0.026X was increased to 0.73X to meet the specificity criterion (Supplementary materials 3: Table S3.1). Increasing the ROC-suggested cutoff for *M. haemolytica* resulted in a slight, but not significant, decrease in median sensitivity for metagenomic sequencing, while for *P. multocida* the increased cutoff resulted in a substantial decrease in sensitivity (Supplementary materials 3: Table S3.1). Increasing the cutoff for metagenomics had minimal impact on the estimates of RPA sensitivity and specificity, with slight improvements in median estimates for RPA performance. For *H. somni*, the ROC-determined theoretical coverage cutoff of 0.02X was used (Supplementary materials 3: Table S3.1), as it resulted in a specificity of 0.91 (95% CrI 0.88–0.94; Table 7). The count of positive samples from FPC and YRL at each time point for *M. haemolytica, P. multocida*, or *H. somni* based on the theoretical coverage cutoff determined by BLCM are summarized in Table 8.

**Table 7:** Estimated sensitivity (Se) and specificity (Sp) of long-read metagenomic sequencing, recombinase polymerase amplification (RPA), and culture (for the detection of *M. haemolytica, P. multocida*, or *H. somni*) or qPCR (for detection of *M. bovis*) based on Bayesian latent class models for each organism in fall-placed calves and yearlings (*n* = 760 deep nasopharyngeal swab samples, *N* = 19 feedlots).

Model outcome	Metric	Method	Median	95% CrI
*M. haemolytica*	Se	Culture	0.62	0.47, 0.85
		Metagenomics^a^	0.63	0.46, 0.84
		RPA	0.31	0.19, 0.45
	Sp	Culture^b^	0.92	0.87, 0.999
		Metagenomics^a^	0.90	0.83, 0.97
		RPA	0.95	0.91, 0.99
*P. multocida*	Se	Culture	0.86	0.75, 0.99
		Metagenomics^a^	0.49	0.39, 0.59
		RPA	0.41	0.32, 0.52
	Sp	Culture^b^	0.97	0.94, 0.999
		Metagenomics^a^	0.90	0.87, 0.93
		RPA	0.98	0.96, 0.999
*H. somni*	Se	Culture	0.58	0.36, 0.84
		Metagenomics^a^	0.49	0.33, 0.66
		RPA	0.52	0.35, 0.69
	Sp	Culture^b^	0.995	0.99, 0.999
		Metagenomics^a^	0.91	0.88, 0.94
		RPA	0.96	0.94, 0.98
*M. bovis*	Se	qPCR	0.96	0.88, 0.999
		Metagenomics (≥2 reads)	0.33	0.26, 0.41
		RPA	0.61	0.53, 0.69
	Sp	qPCR	0.998	0.99, 0.999
		Metagenomics (≥2 reads)	0.97	0.94, 0.98
		RPA	0.92	0.89, 0.95

CrI, credible interval.

^a^Metagenomics result classified as positive based on theoretical coverage of >0.29X for *M. haemolytica*, >0.73X for *P. multocida*, and >0.02X for *H. somni*.

^b^Prior for culture specificity—beta(100,1.5); based on most likely value = 0.995, and 95% sure > 0.975.

**Table 8:** Number (%) of samples classified as positive for detection of *M. haemolytica, P. multocida*, or *H. somni* based on theoretical coverage cutoffs determined by Receiver Operating Characteristic analysis followed by Bayesian latent class analysis in fall-placed calves (FPC) and yearlings (YRL) at and shortly after feedlot arrival (*n* = 760 deep nasopharyngeal swab samples, *N* = 19 feedlots).

Bacteria	Theoretical coverage cutoff (×)	FPC		YRL	
		Arrival	14 DOF	Arrival	14 DOF
*M. haemolytica*	0.29	82 (32%)	56 (22%)	17 (14%)	37 (31%)
*P. multocida*	0.73	65 (25%)	30 (12%)	28 (23%)	22 (18%)
*H. somni*	0.02	22 (8.5%)	47 (18%)	10 (8.3%)	16 (13%)

DOF, days on feed.

**Figure 2:**
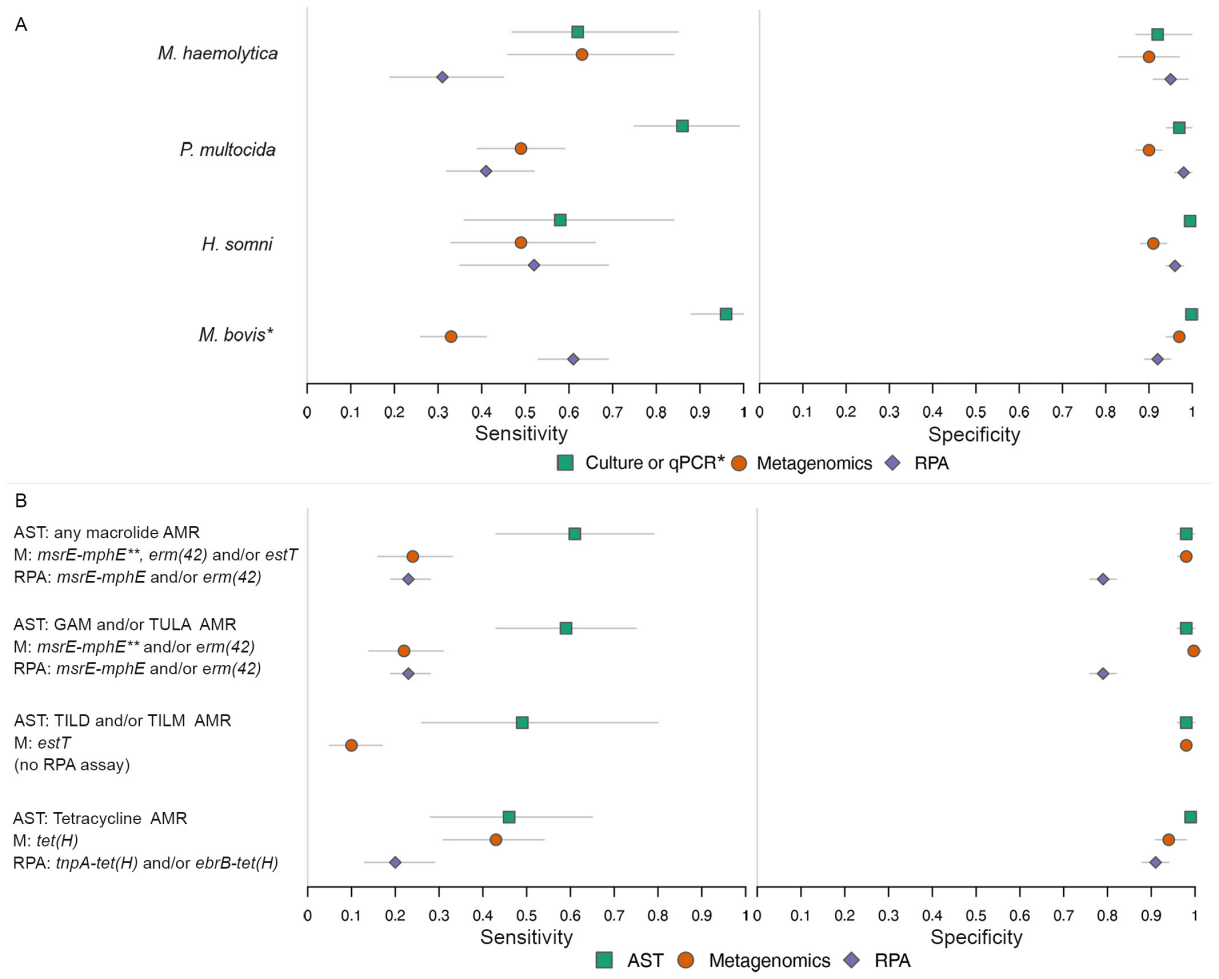
Estimated diagnostic sensitivity and specificity for detection from Bayesian latent class models. **(A)** (Top): detection of *M. haemolytica, P. multocida, H. somni*, or *M. bovis*. **(B)** (Bottom): antimicrobial resistance (AMR) and resistance genes in *M. haemolytica, P. multocida*, or *H. somni*. Bars represent the 95% credible intervals. AST, antimicrobial susceptibility testing; AMR, antimicrobial resistance; M, metagenomics; RPA, recombinase polymerase amplification; GAM, gamithromycin; TULA, tulathromycin; TILD, tildipirosin; TILM, tilmicosin. ^*^Estimates for *M. bovis* are for qPCR, whereas all other organisms are for culture. ^**^msrE-mphE—includes any samples positive for *msrE* or *mphE*.

The BLCM estimates for sensitivity for detection of *M. haemolytica, P. multocida*, or *H. somni* were generally low for all tests (< 65%) except for *P. multocida* culture (Table 7 and Figure 2). For *M. haemolytica*, the sensitivities of culture and metagenomics were comparable, but that of RPA was significantly lower with non-overlapping credible intervals. In contrast, for *P. multocida* the sensitivity was highest for culture, while those of metagenomics and RPA were comparable but significantly lower. For *H. somni*, the estimates for sensitivity were similar for all tests.

Estimates for BLCM specificities for all tests were higher (Table 7 and Figure 2). For *M. haemolytica*, the credible intervals overlapped for all tests. For *P. multocida*, the specificities of culture and RPA were comparable, and both were significantly higher than the specificity of metagenomics. For *H. somni*, the specificity of culture was significantly greater than that of both metagenomics and RPA. When comparing the performance metrics for each test across bacterial species, all credible intervals for sensitivity and specificity overlapped. Individual tests were not significantly better at detecting one type of bacteria as compared to the others.

When covariance was incorporated between culture and metagenomics, the estimates for sensitivity and specificity for all tests were similar to models without the additional covariance terms, with substantial overlap in credible intervals in each of the bacterial models (Supplementary materials 3: Table S3.2). In addition, the credible intervals for covariance between culture and metagenomics included zero. Together these findings suggested that the additional terms were not important in these models and the more parsimonious models without covariance between culture and metagenomics were reported.

Serially excluding each population (FPC vs. YRL and arrival vs. 14 DOF) did not substantially change estimates for test performance for the detection of *M. haemolytica*, with overlap in the credible intervals for each metric across all models (Supplementary materials 3: Table S3.3).

Among the library preparation controls, the median total read length was 3,829 base pairs (bp; IQR 0–20,487) for *M. haemolytica*, 4,685 bp (IQR 0–23,218) for *P. multocida*, and 0 bp (IQR 0–0) for *H. somni*. While adjusting total read lengths for the mean control read lengths impacted the ROC-determined baseline theoretical coverage cutoffs, adjusting for the median control read lengths had no impact on the ROC-determined cutoffs (Supplementary materials 3: Table S3.4). These cutoffs did not meet the threshold of 0.90 for metagenomics specificity. When adjusted to 0.29X coverage for *M. haemolytica* and 0.73X coverage for *P. multocida* for direct comparison to the models for unadjusted data, the specificity threshold was met for both mean- and median-adjusted data, and the estimated test performance was very similar for both adjusted and unadjusted data. This suggested that adjustment for reads present in library preparation controls had little impact on the final cutoffs or BLCM results (Supplementary materials 3: Table S3.4) justifying the use of unadjusted data in primary analyses.

### 3.2 Bayesian latent class models: detection of samples with AMR in target bacteria (AST and metagenomics) or in samples with target bacteria (RPA)

In the models comparing AST, metagenomics, and RPA for the detection of macrolide AMR/ARGs, AST consistently exhibited higher sensitivity than metagenomics or RPA, although sensitivity for detecting phenotypic AMR or ARGs was generally low for all tests (Table 9 and Figure 2). Specificities were higher and similar for AST and metagenomics in the macrolide resistance models with overlapping credible intervals, except for the model comparing any phenotypic macrolide resistance by AST, detection of *msrE-mphE* and/or *erm(42)* by RPA, and any gene associated with macrolide resistance detected by metagenomics. The specificity of RPA for detecting macrolide resistance within samples positive for the target organisms was consistently lower than that of both AST and metagenomics for detecting AMR in the target organisms (Table 9 and Figure 2). The metagenomic data for the samples that were negative for both AST and metagenomics and positive for RPA were examined in more detail, identifying eight samples with *Klebsiella pneumoniae* reads that contained *msrE-mphE* and one sample with *E. coli* reads that contained *msrE-mphE*.

**Table 9:** Estimated sensitivity (Se) and specificity (Sp) from Bayesian latent class models comparing culture/antimicrobial susceptibility testing (AST^a^), long-read metagenomic sequencing, and recombinase polymerase amplification (RPA) for detection of antimicrobial resistance in *M. haemolytica, P. multocida*, or *H. somni* in fall-placed calves and yearlings (*n* = 760 deep nasopharyngeal swab samples, *N* = 19 feedlots).

Model^b^	Metric	Method	Median	95% CrI
AST^a^: GAM, TILD, TILM, and/or TULA resistance Metagenomics: *msrE*−*mphE*^c^*, erm(42)*, and/or *estT* RPA: *msrE-mphE* and/or *erm(42)* (*n =* 758)	Se	AST	0.61	0.43, 0.79
		Metagenomics	0.24	0.16, 0.33
		RPA	0.23	0.19, 0.28
	Sp	AST	0.98	0.96, 0.999
		Metagenomics	0.98	0.96, 0.99
		RPA	0.79	0.76, 0.82
AST^a^: GAM, TILD, TILM, and/or TULA resistance Metagenomics: any ARG potentially impacting resistance to macrolide class RPA: *msrE-mphE* and/or *erm(42)* (*n =* 758)	Se	AST	0.86	0.66, 0.999
		Metagenomics	0.35	0.25, 0.45
		RPA	0.24	0.20, 0.30
	Sp	AST	0.98	0.95, 0.999
		Metagenomics	0.91	0.88, 0.93
		RPA	0.79	0.76, 0.82
AST^a^: GAM and/or TULA resistance Metagenomics: *msrE*−*mphE*^c^ and/or *erm(42)* RPA: *msrE-mphE* and/or *erm(42)* (*n =* 758)	Se	AST	0.59	0.43, 0.75
		Metagenomics	0.22	0.14, 0.31
		RPA	0.23	0.19, 0.28
	Sp	AST	0.98	0.96, 0.999
		Metagenomics	0.997	0.99, 0.999
		RPA	0.79	0.76, 0.82
AST^a^: TILD and/or TILM resistance Metagenomics: *estT* (no comparable RPA assay) (*n =* 758)	Se	AST	0.49	0.26, 0.80
		Metagenomics	0.10	0.05, 0.17
	Sp	AST	0.98	0.96, 0.999
	Metagenomics	0.98	0.97, 0.99
AST^a^: Tetracycline resistance Metagenomics: *tet(H)* RPA: ICE *tnpA-tet(H)* and/or *ebrB-tet(H)* (*n =* 760)	Se	AST	0.46	0.28, 0.65
		Metagenomics	0.43	0.31, 0.54
		RPA	0.20	0.13, 0.29
	Sp	AST	0.99	0.98, 0.999
		Metagenomics	0.94	0.91, 0.98
		RPA	0.91	0.88, 0.94
AST^a^: Tetracycline resistance Metagenomics: any ARG potentially impacting resistance to tetracycline class RPA: ICE *tnpA-tet(H)* and/or *ebrB-tet(H)* (*n =* 760)	Se	AST	0.47	0.28, 0.73
		Metagenomics	0.50	0.38, 0.62
		RPA	0.21	0.12, 0.30
	Sp	AST	0.99	0.98, 0.999
		Metagenomics	0.82	0.78, 0.87
		RPA	0.91	0.89, 0.94

CrI, credible interval; GAM, gamithromycin; TULA, tulathromycin; TILD, tildipirosin; TILM, tilmicosin (CLSI breakpoint only available for *M. haemolytica*); ICE, integrative and conjugative element-associated target variant.

^a^For AST, intermediate MICs were classified as susceptible.

^b^Any model containing macrolide resistance included 758 samples on account of macrolide results not being available for two RPA samples due to insufficient volume for testing.

^c^*msrE-mphE*—includes any samples positive for *msrE* or *mphE*.

The various configurations of phenotypic macrolide resistance and ARGs included in the three-test models (models that compare AST, metagenomics, and RPA) had no significant impact on test performance for most models (Table 9 and Figure 2). The credible intervals for AST sensitivity and specificity overlapped among all macrolide model configurations. Similarly, sensitivity and specificity estimates for RPA were very similar across model configurations. The credible intervals for estimates of sensitivity of metagenomics overlapped between most models; however, the specificity of metagenomics was significantly lower for the model including all detected genes reported by the CARD (Alcock et al., 2023) to be associated with macrolide resistance than in any models limited to *msrE-mphE, erm(42)*, or *estT* (Table 9 and Figure 2).

The sensitivity for metagenomics was significantly lower in the two-test model comparing metagenomic detection of *estT* to phenotypic resistance to TILD or TILM than in the three-test model comparing metagenomic detection of any macrolide resistance gene with the detection of any phenotypic macrolide resistance by AST (Table 9 and Figure 2).

For the models comparing detection of *tet(H)* by metagenomics to detection of phenotypic tetracycline resistance by AST and detection *tet(H)* within ICE-associated target variants *tnpA-tet(H)* or *ebrB-tet(H*) by RPA, the sensitivities of AST and metagenomics were comparable, while the sensitivity of RPA was lower than that of metagenomics (Table 9 and Figure 2). The specificity of AST was higher than either metagenomics or RPA, which were comparable to each other. A similar pattern in test sensitivities was evident in the model comparing detection of any tetracycline resistance gene by metagenomics to detection by AST and detection of ICE-associated target variants by RPA. In this model the specificity of AST was higher than that of either metagenomics or RPA, while the specificity of RPA was higher than that of metagenomics (Table 9 and Figure 2).

The sensitivities and specificities of AST and RPA remained consistent between the separate configurations of tetracycline resistance models. The sensitivities of metagenomic detection of either *tet(H)* or any tetracycline resistance gene were similar across model configurations, but the specificity of metagenomics for the detection of any tetracycline resistance gene was lower than the specificity for the detection of *tet(H)* (Table 9 and Figure 2).

The model for florfenicol resistance detected by AST and metagenomics suggested low sensitivity for both tests, while specificity estimates were higher (Supplementary materials 3: Table S3.5). However, given the very low prevalence of florfenicol resistance detected by AST in this study (Table 2), these results should be interpreted with caution.

The impact of grouping isolates classified as intermediate by AST as resistant rather than susceptible was minimal (Supplementary materials 3: Table S3.5), particularly for the macrolide AMR models. For the tetracycline AMR models there was a trend toward increased sensitivity for AST when intermediate results were classified as resistant compared to models in which they were classified as susceptible, but the differences were not significant. Similarly, there was very little change in the estimates for metagenomics or RPA.

### 3.3 Bayesian latent class models: integrative and conjugative element-associated targets

In a separate model examining the detection of the ICE-associated target variants, sensitivity estimates were low both for metagenomics (0.49, 95% CrI 0.22–0.96) in detecting genes identified on reads of *Pasteurellaceae* bacteria and for RPA for detecting ICE-associated target variants (0.14, 95% CrI 0.09–0.25) in samples where the bacteria had also been detected. The specificities were substantially higher than sensitivity for both tests; with the estimated specificity of metagenomics (0.99, 95% CrI 0.97–0.999) being higher than that of RPA (0.90, 95% CrI 0.87–0.93).

### 3.4 Bayesian latent class models: *M. bovis*

A cutoff of ≥2 reads was required to obtain a specificity ≥0.90 for metagenomic detection of *M. bovis*. In the model comparing qPCR, metagenomics (≥2 reads), and RPA for the detection of *M. bovis*, the estimated sensitivities were significantly different, with qPCR being greater than RPA, which was greater than metagenomics. The specificity of qPCR was also higher than that of metagenomics and RPA, which were comparable to each other (Table 7).

When the metagenomic long-read sequencing results were classified using the ≥2 read cutoff, 11/260 (4.2%) FPC were classified as positive for *M. bovis* by metagenomics on arrival, while 53/260 (20%) were positive at 14 DOF. Of the YRL, 0/120 were positive on arrival, and 13/120 (11%) were positive at 14 DOF.

### 3.5 Patterns of multiple organism detection across tests

Single BRD organisms were detected in 309/760 (40.7%) samples by culture or qPCR, 222/760 (29.2%) samples by sequencing, and 219/760 (28.8%) samples by RPA (Supplementary materials 4: Table S4.1). Two or more BRD organisms were detected in 125/760 (16.4%) samples by culture or qPCR, 118/760 (15.5%) samples by sequencing; and 72/760 (9.5%) samples by RPA, with only sequencing (10/760, 1.3%) and RPA (10/760, 1.3%) detecting all four BRD organisms in a sample. There were no BRD organisms detected in 326/760 (42.9%) samples by culture or qPCR, 420/760 (55.3%) samples by sequencing, and 469 (61.7%) samples by RPA.

### 3.6 Bayesian latent class models: *M. haemolytica* serotypes

In the two-test model comparing long-read metagenomic sequencing targeting genomic regions associated with *M. haemolytica* serotypes A1 and A6 and detection of *M. haemolytica* by RPA targeting serotypes A1 and A6, the estimated sensitivities and specificities were similar for both tests. While the median sensitivities of both metagenomics (0.23, 95% CrI 0.11–0.51) and RPA (0.27, 95% CrI 0.13–0.59) were numerically lower than estimates from the three-test *M. haemolytica* model with culture, metagenomics for all serotypes, and RPA for serotypes A1 and A6 (Table 7), the estimates were not significantly different. In contrast, the specificities of both metagenomics (0.97, 95% CrI 0.93–0.999) and RPA (0.96, 95% CrI 0.92–0.999) for detection of serotypes A1 and A6 were numerically but not significantly higher than all estimates for the three-test *M. haemolytica* model (Table 7).

### 3.7 Bayesian latent class models: impact of sample transit time

Analysis of the subset of samples with a transit time to the diagnostic laboratory within two days of sample collection had very little impact on estimates from BLCM (Supplementary materials 3: Table S3.6). Changes to median estimates were small with no differences between models using the two-day transit time subset compared to the full dataset, and there were no consistent trends toward increased test performance with shorter transit times among the models examined.

### 3.8 Positive and negative predictive values for detection of metagenomic ARGs and AST detection of AMR and detection of *M. haemolytica*

**Figure 3:**
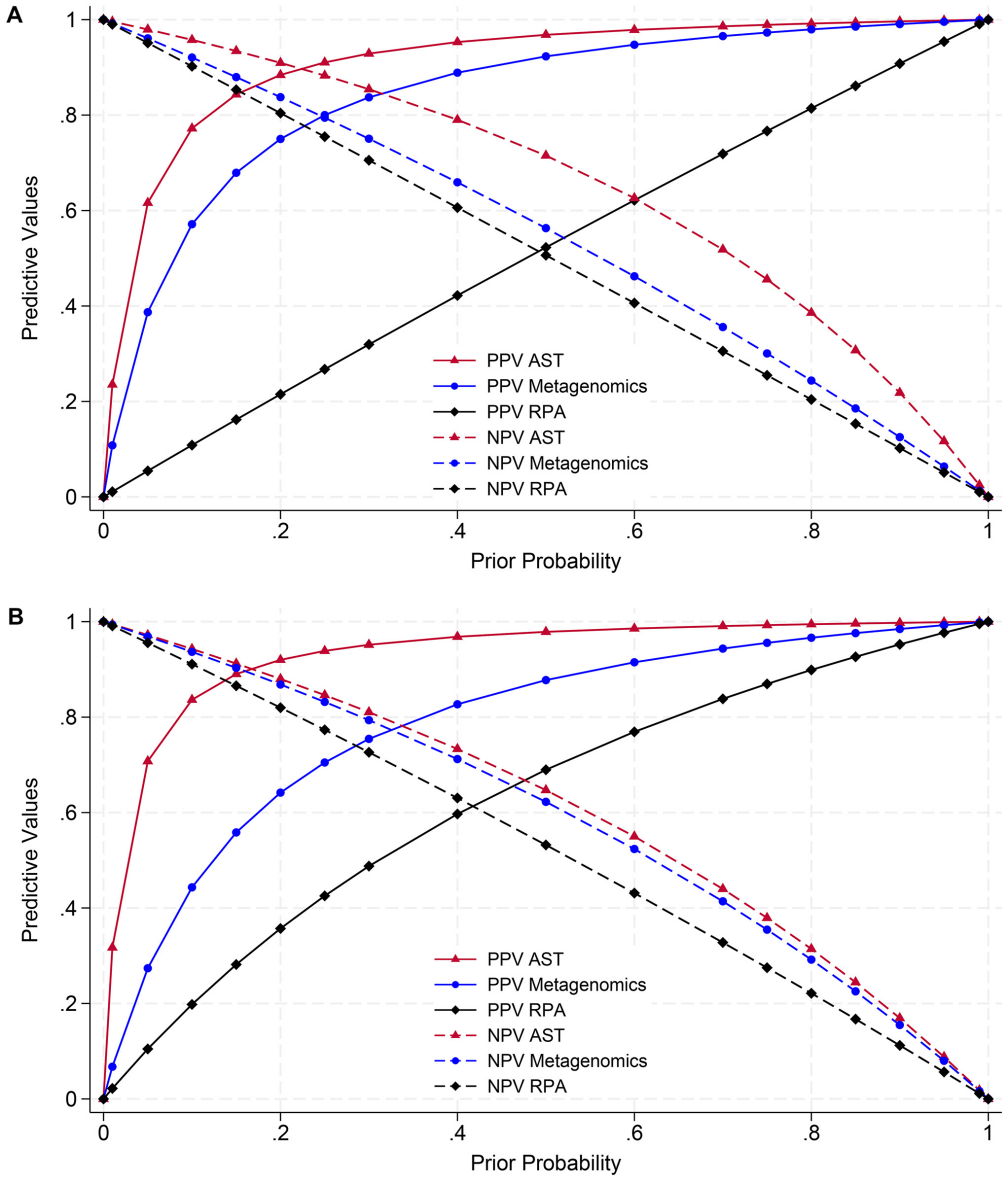
Positive and negative predictive values (PPVs and NPVs) for detection of *M. haemolytica, P. multocida*, or *H. somni* with macrolide or tetracycline resistance. Estimates are based on sensitivities and specificities from Bayesian latent class models (Table 9). **(A)** (Top): detection of resistance to gamithromycin, tildipirosin, tilmicosin, and/or tulathromycin by antimicrobial susceptibility testing (AST), *msrE-mphE, erm(42)*, and/or *estT* by long-read metagenomics, or *msrE-mphE* or *erm(42)* by recombinase polymerase amplification (RPA). **(B)** (Bottom): detection of resistance to tetracycline by culture and AST, *tet(H)* by long-read metagenomics, or *tet(H)* as part of an integrative conjugative element (ICE)-associated target variant by RPA.

Based on results of the BLCMs (Table 9), estimates of the PPV for the detection of macrolide ARGs by metagenomics or RPA, or resistance to any macrolide, in any of *M. haemolytica, P. multocida*, or *H. somni* exceeded 75% when the prior probability of detection exceeded 9% for AST, 20% for metagenomics, and 75% for RPA (Figure 3; Supplementary materials 5: Table S5.1). The estimates of NPV exceeded 75% when the prior probability was less than 46% for AST, 30% for metagenomics, or 25% for RPA. For detection of tetracycline ARGs by metagenomics, ICE-associated target variants by RPA, and tetracycline resistance by AST, in any of the three organisms, estimates of PPV exceeded 75% when the prior probability was greater than 6% for AST, 30% for metagenomics, and 60% for RPA (Figure 3; Supplementary materials 5: Table S5.2). The estimates of NPV exceeded 75% when the prior probability was less than 38% for AST and 35% for metagenomics or RPA.

**Figure 4:**
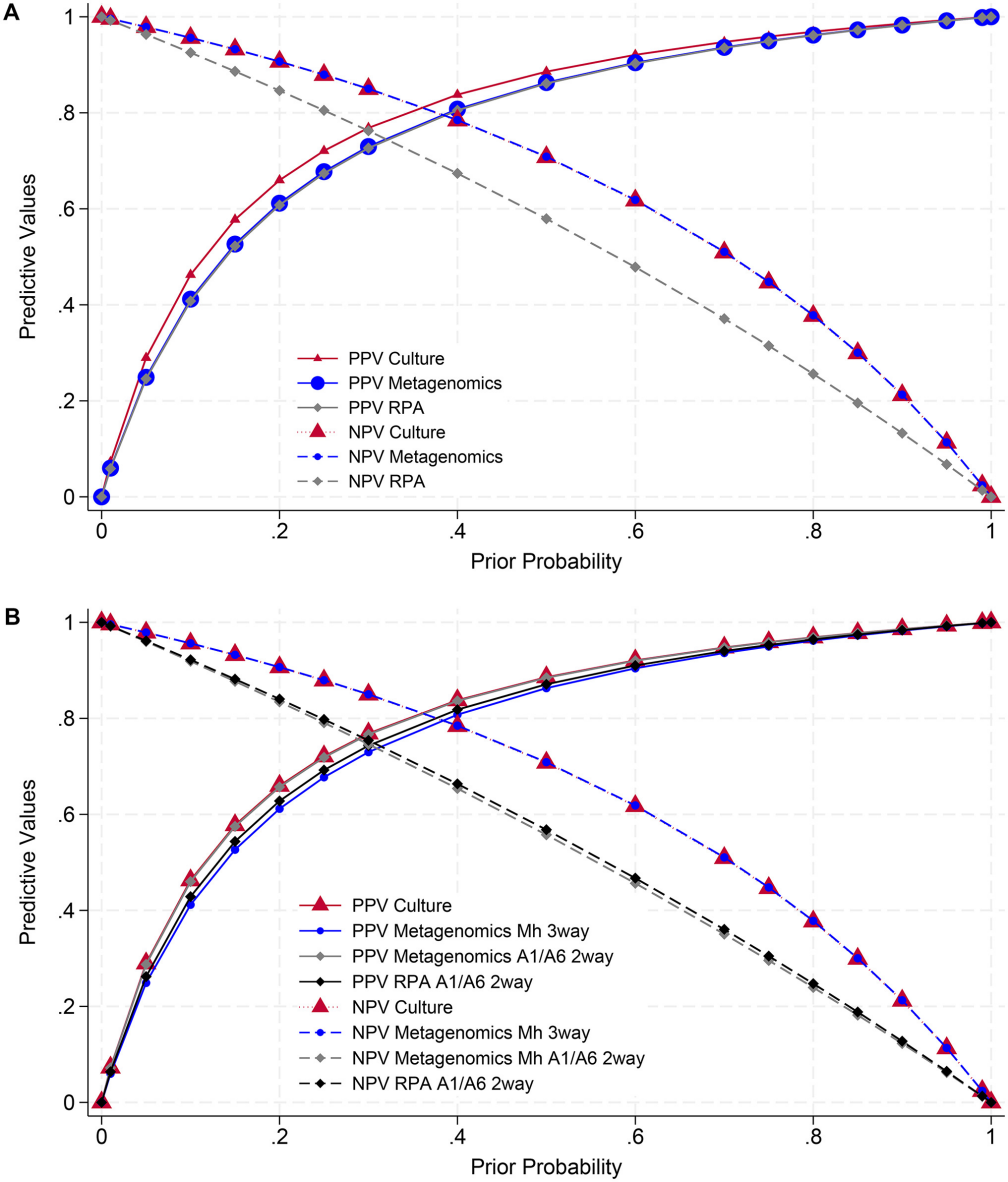
Positive and negative predictive values (PPVs and NPVs) for the detection of *M. haemolytica* (Mh) from culture, long-read metagenomics, or recombinase polymerase amplification (RPA). Estimates are based on sensitivities and specificities from Bayesian latent class models (Table 7). **(A)** (Top): models with three-way test comparisons for any *M. haemolytica* (culture or metagenomics) or serotypes A1/A6 (RPA). **(B)** (Bottom): models with three-way test comparisons for all *M. haemolytica* (culture or metagenomics, RPA excluded) and two-way comparisons for serotypes A1/A6 (metagenomics and RPA only).

Estimates of the PPV and NPV for detection of *M. haemolytica* by metagenomics, culture, or RPA had little variation, depending on the models for three-way or two-way test comparisons (Figure 4). Three-way comparisons included detection of any *M. haemolytica* serotype by metagenomics and culture, plus detection by RPA, which only targets serotypes A1 and A6, and found comparable PPVs for all three tests, whereas the NPV was lower for RPA compared to metagenomics or culture. Conversely, two-way model comparisons looked specifically at the detection of serotypes A1 and A6 by metagenomics or RPA. The PPVs were comparable for detection of any serotype (metagenomics or culture) and of serotypes A1/A6 (metagenomics or RPA), whereas the NPV was better for any serotype (metagenomics or culture) compared to tests specific for serotypes A1/A6 (metagenomics or RPA).

### 3.9 Differences over time and between FPC and YRL

Detection of *M. haemolytica* at 14 DOF as compared to arrival processing and between FPC and YRL generally did not differ for culture, metagenomics, or RPA testing (Table 10), with two exceptions. First, metagenomics was more likely to detect any *M. haemolytica* serotype in YRL at 14 DOF compared to at arrival (OR 2.71, 95% CI 2.00–3.67). Secondly, *M. haemolytica* serotypes A1 and A6 were more likely to be detected in FPC vs. YRL at arrival (OR 4.89, 95% CI 1.40–17.1) using RPA. The same difference between FPC and YRL at arrival was observed when the metagenomic analysis of *M. haemolytica* was limited to serotypes A1 and A6 (OR 4.26, 95% CI 1.52–12.0; Table 10).

**Table 10:** Differences in detection of important bovine respiratory disease bacteria using culture, long-read metagenomics, and recombinase polymerase amplification (RPA) among samples collected at arrival and 14 days on feed (DOF) for fall-placed calves (FPC) and yearlings (YRL) after accounting for transit time (*n* = 760 deep nasopharyngeal swab samples, *N* = 19 feedlots).

Bacteria + Test	FPC: 14DOF vs. arrival			YRL: 14DOF vs. arrival			FPC vs. YRL: arrival			FPC vs. YRL: 14 DOF		
	OR	95% CI	*P*	OR	95% CI	*P*	OR	95% CI	*P*	OR	95% CI	*P*
*M. haemolytica*											
Culture	1.13	(0.64, 2.00)	0.67	15.6-10,-1.3137pt1.56	(0.65, 3.76)	0.32	1.03	(0.43, 2.52)	0.94	0.75	(0.25, 2.25)	0.61
Metagenomics^a^	0.57	(0.25, 1.30)	0.18	**2.71**	**(2.00, 3.67)**	**0.001**	15.6-10,-1.3137pt2.86	(0.98, 8.33)	0.054	0.60	(0.19, 1.89)	0.39
RPA (serotypes A1 and A6)	0.49	(0.19, 1.29)	0.15	4.03	(1.00, 16.3)	0.051	15.6-10,-1.3137pt**4.89**	**(1.40, 17.1)**	**0.01**	0.60	(0.19, 1.87)	0.38
Metagenomics serotypes A1 and A6^b^	0.38	(0.14, 1.06)	0.07	3.33	(0.93, 11.9)	0.06	4.26	**(1.52, 12.0)**	**0.006**	0.49	(0.10, 2.33)	0.37
*P. multocida*												
Culture	**0.30**	**(0.14, 0.67)**	**0.003**	0.66	(0.26, 1.66)	0.38	**2.48**	**(1.02, 6.01)**	**0.045**	1.13	(0.57, 2.26)	0.72
Metagenomics^a^	**0.42**	**(0.26, 0.68)**	**0.001**	0.74	(0.22, 2.45)	0.62	1.06	(0.32, 3.52)	0.93	0.60	(0.20, 1.81)	0.36
RPA	**0.17**	**(0.06, 0.47)**	**0.001**	0.75	(0.24, 2.35)	0.62	2.18	(0.95, 5.00)	0.07	20.6-10,-12143pt0.51	(0.19, 1.35)	0.18
*H. somni*												
Culture	1.74	(0.59, 5.12)	0.31	15.6-10,-1.3137pt1.00	(0.06, 17.7)	0.99	7.28	(0.96, 55.4)	0.06	**12.7**	**(1.71, 93.9)**	**0.01**
Metagenomics^a^	2.20	(0.97, 5.02)	0.06	**1.70**	**(1.28, 2.25)**	**0.001**	1.06	(0.37, 3.04)	0.91	1.38	(0.53, 3.60)	0.52
RPA	47.8-10,-12137pt0.71	(0.28, 1.78)	0.46	1.18	(0.64, 2.16)	0.60	2.47	(0.77, 7.95)	0.13	1.48	(0.65, 3.39)	0.35
*M. bovis*												
qPCR	**21.3**	**(7.4, 61.4)**	**0.001**	15.6-10,-1.3137pt2.64	(0.86, 8.08)	0.09	0.25	(0.06, 1.14)	0.07	15.6-10,-1.3143pt2.03	(0.83, 5.01)	0.12
Metagenomics^c, d^	**6.16**	**(2.96, 14.0)**	**0.001**	**20.2**	**(3.31**, ***∞*****)**	**0.001**	6.16	(0.97, ***∞***)	0.054	**2.02**	**(1.02, 4.23)**	**0.04**
RPA	**3.86**	**(2.04, 7.32)**	**0.001**	1.98	(0.45, 8.69)	0.24	0.76	(0.24, 2.38)	0.42	1.49	(0.71, 3.13)	0.37

OR, odds ratio; 95% CI, 95% confidence interval; *P, P*-value.

^a^Metagenomics result classified as positive based on theoretical coverage of >0.29X for *M. haemolytica*, >0.73X for *P. multocida*, and >0.02X for *H. somni*.

^b^Classified as positive if ≥1 read of *M. haemolytica* identified as serotype.

^c^Classified as positive ≥2 reads.

^d^Exact logistic regression.

Cells with statistically significant odds ratios (*P* ≤ 0.05) are highlighted in bold and with gray shading.

*P. multocida* was detected less often in FPC at 14 DOF vs. at arrival for all three tests (Table 10). The frequency of *P. multocida* generally did not vary for any other time or age comparisons within tests, with the exception that *P. multocida* was more likely to be detected in FPC than YRL by culture at arrival (OR 2.48, 95% CI 1.02–6.01; Table 10).

The only two differences among sampling times and age groups for *H. somni* (Table 10) were that culture was more likely to detect *H. somni* in FPC vs. YRL at 14 DOF (OR 12.7, 95% CI 1.71–93.9) and metagenomics was more likely to detect *H. somni* in YRL at 14 DOF as compared to arrival processing (OR 1.70, 95% CI 1.28–2.25).

*M. bovis* was more likely to be detected at 14 DOF than at arrival processing in FPC for all three tests (qPCR, metagenomics, and RPA; Table 10). No other time or age differences were significant for qPCR or RPA. However, metagenomics detected *M. bovis* more often at 14 DOF than at arrival processing for YRL and in FPC as compared to YRL at 14 DOF.

The differences among tests in macrolide resistance among sample times and age groups varied more frequently than for the other metrics examined (Table 11). The likelihood of detecting macrolide resistance was higher in FPC at 14 DOF compared to at arrival for both AST and metagenomics. Whereas, RPA detected macrolide resistance more frequently in FPC at arrival than at 14 DOF and in FPC than YRL at arrival (Table 11). Macrolide resistance was identified more often by AST in FPC as compared to YRL at 14 DOF. This same difference was not observed based on detection of *msrE-mphE* and/or *erm(42)* by either metagenomics or RPA. This difference was, however, consistent with that detected by metagenomics for *estT* on the target bacterial reads (Table 11). Age group and sample time differences for 15-membered (gamithromycin and tulathromycin) and 16-membered ring (tildipirosin and tilmicosin) macrolides detected by AST did not differ from those observed for overall resistance or from each other (Table 11).

**Table 11:** Differences in detection of macrolide resistance^a^, tetracycline resistance, or integrative and conjugative elements in samples containing *M. haemolytica, P. multocida*, or *H. somni* using antimicrobial susceptibility testing (AST), long-read metagenomics, and recombinase polymerase amplification (RPA) among samples collected at arrival and 14 days on feed (DOF) for fall-placed calves (FPC) and yearlings (YRL) after accounting for transit time (*n* = 760 deep nasopharyngeal swab samples, *N* = 19 feedlots).

	FPC: 14DOF vs. arrival			YRL: 14DOF vs. arrival			FPC vs. YRL: arrival			FPC vs. YRL: 14 DOF		
	51.4-10,-12138pt**OR**	**95% CI**	***P***	**OR**	**95% CI**	***P***	**OR**	**95% CI**	***P***	51.4-10,-12143pt**OR**	**95% CI**	***P***
Macrolide resistance—culture and AST based on CLSI breakpoints												
AST—any macrolides	21.6	(10.7, 43.5)	0.001	2.26	(0.74, 6.90)	0.15	0.64	(0.13, 3.09)	0.58	6.12	(1.24, 30.2)	0.03
AST—GAM and/or TULA	20.8	(10.2, 41.9)	0.001	2.27	(0.74, 6.91)	0.15	0.6	(0.12, 2.97)	0.53	5.49	(1.14, 26.3)	0.03
AST—TILM^b^ and/or TILD	19.5	(7.45, 50.9)	0.001	2.75	(0.61, 12.4)	0.19	0.61	(0.10, 3.63)	0.59	4.33	(1.00, 18.8)	0.050
Macrolide resistance genes—long-read metagenomic sequencing ≥1 read of *M. haemolytica*, *P. multocida,* or *H. somni* with ARG												
±*msrE* ±*mphE* ±*erm*(42)^c^	57.0	(9.65, *∞*)	0.001	3.66	(0.68, 37.1)	0.17	0.23	(0.00, 2.95)	0.25	15.6-10,-1.3143pt0.11	(0.88, 5.97)	0.33
*estT* ^c^	20.6-10,-12138pt 2.56	(0.96, 7.69)	0.06	1.00	(0.03, ***∞***)	0.99	20.6-10,-12137pt5.50	(0.81, ***∞***)	0.09	9.67	(1.47, 412)	0.01
Macrolide resistance genes—RPA positive in samples positive for *M. haemolytica*, *P. multocida,* or *H. somni*												
±*msrE-mphE* ±*erm(42)*	0.32	(0.14, 0.71)	0.005	1.76	(0.74, 4.20)	0.20	3.05	(1.43, 6.52)	0.004	0.55	(0.25, 1.25)	0.15
Tetracycline resistance—culture and AST based on CLSI breakpoints												
AST—Tetracycline	32.9	(7.72, 140)	0.001	20.6-10,-12137pt2.94	(0.34, 25.2)	0.33	20.6-10,-12137pt0.23	(0.03, 1.63)	0.14	20.6-10,-12143pt2.57	(0.59, 11.1)	0.21
Tetracycline resistance genes—long-read metagenomic sequencing (≥1 read of *M. haemolytica*, *P. multocida,* or *H. somni* with ARG)												
*tet(H)*	2.81	(1.24, 6.34)	0.013	4.20	(1.89, 9.35)	0.001	6.71	(1.61, 28.0)	0.009	4.48	(1.77, 11.4)	0.002
ICE-associated target variants with long-read metagenomic sequencing in ≥1 read of *M. haemolytica*, *P. multocida,* or *H. somni*												
*tnpA-tet(H)* and/or *ebrB-tet(H)*	2.84	(1.01, 7.94)	0.047	7.37	(0.93, 58.3)	0.06	13.7	(1.72, 109)	0.01	5.29	(2.1, 13.3)	0.001
ICE-associated target variants with RPA in samples positive for *M. haemolytica*, *P. multocida,* or *H. somni*												
*tnpA-tet(H)* and/or *ebrB-tet(H)*	0.84	(0.26, 2.73)	0.78	2.18	(0.59, 8.00)	0.24	1.59	(0.52, 4.89)	0.42	0.62	(0.21, 1.78)	0.37

OR, odds ratio; 95% CI, 95% confidence interval; *P, P*-value; GAM, gamithromycin; TULA, tulathromycin; TILM, tilmicosin; TILD, tildipirosin; ICE, integrative conjugative element.

^a^Any model containing macrolide resistance included 758 samples on account of macrolide results not being available for two RPA samples due to insufficient volume for testing.

^b^TILM CLSI breakpoints not available for *P. multocida* and *H. somni*—AST only available for *M. haemolytica*.

^c^Exact logistic regression.

Cells with statistically significant odds ratios (*P* ≤ 0.05) are highlighted in bold and with gray shading.

For tetracycline resistance detection in all organisms, all time and age comparisons were significant for the detection of *tet(H)* by metagenomics (Table 11); 14 DOF was higher than arrival for both FPC and YRL and detection was more likely in FPC than YRL at both time points. Comparatively, when considering AST, tetracycline resistance only varied between 14 DOF and at arrival processing for FPC (OR 32.9, 95% CI 7.71–140).

Similar to what was observed for macrolide resistance genes, there were substantial differences in comparisons of detection of ICE-associated target variants between metagenomics and RPA across time periods and age groups (Table 11). There were no significant differences for any comparison using RPA. Conversely, ICE-associated target variants were more likely to be detected by metagenomics in BRD bacteria reads from FPC than YRL at both arrival and 14 DOF and were detected more often at 14 DOF than arrival FPC. The difference between 14 DOF vs. at arrival comparison for YRL also trended toward significance (OR 7.37, 95% CI 0.93–58.3; Table 11).

## 4 Discussion

This study demonstrated successful application of a recently developed, long-read metagenomic sequencing protocol in a commercial diagnostic laboratory for the detection of BRD bacteria and ARGs in feedlot cattle from western Canadian commercial feedlots. Collaboration with a national surveillance program for antimicrobial use (AMU)/AMR in feedlot cattle (Hannon et al., 2020; CFAASP, 2022, 2025) enabled access to a large, geographically distributed sample set, along with culture, and AST results from samples collected by private veterinary clinics. Application of BLCMs was used to compare the performance of the long-read metagenomics protocol with recently developed RPA assays and conventional culture/AST for the detection of *Pasteurellaceae* and associated AMR, and qPCR for *M. bovis*, without assuming that conventional culture and AST or qPCR represented a gold standard. This work demonstrates the potential for large scale implementation of long-read metagenomic sequencing to support antimicrobial stewardship and AMR surveillance initiatives in the agriculture industry. These comparisons were made by defining AMR as the detection of either phenotypic or genotypic AMR. Future work is required to understand the linkages between detection of ARGs, gene expression, and phenotypic AMR.

This study also assessed differences in detection of BRD bacteria and associated AMR in FPC vs. YRL at different times early in the feeding period. Fall-placed calves are managed differently than YRLs in western Canadian feedlots owing to their higher risk of clinical BRD (Taylor et al., 2010; Andrés-Lasheras et al., 2021; Otto et al., 2024). Consistent with this difference, detection of *M. haemolytica* serotypes A1/A6 by both long-read metagenomics and RPA was more likely in FPC vs. YRL on arrival. Conversely, the odds of any *M. haemolytica* detection did not differ across age groups. While serotypes A1/A6 are more frequently associated with clinical BRD (Klima et al., 2014), serotype information is not typically available to practitioners that rely on traditional culture of *M. haemolytica*. In the present study serotypes A1/A6 were less prevalent based on metagenomics than A2, similar to a previous report by Andrés-Lasheras et al. (2021).

Metagenomics most commonly identified ARGs for tetracyclines, macrolides, sulfonamides, florfenicol, and aminoglycosides. While ARGs associated with aminoglycoside resistance and *sul2* were relatively frequent, they were not considered for further analysis. Treatment and management of BRD in Canadian feedlot cattle primarily relies on macrolides, tetracyclines, and florfenicol (Brault et al., 2019; CFAASP, 2023). Aminoglycosides are not used in feedlot cattle (Brault et al., 2019; CFAASP, 2023). Sulfonamide antimicrobials are typically used in feedlot cattle in combination with trimethoprim (CFAASP, 2023). Only 12 samples were identified with ARGs associated with trimethoprim resistance.

*P. multocida* was more likely to be detected in FPC on arrival vs. 14 DOF regardless of whether using culture, metagenomics, or RPA. Our previous work also detected *P. multocida* more frequently by culture or metagenomics at arrival than at 13 DOF in FPC receiving tulathromycin metaphylaxis, but not for those receiving oxytetracycline metaphylaxis (Abi Younes et al., 2024, 2025). While no information on metaphylaxis was available in the present study, macrolides, including tulathromycin, are commonly used for injectable metaphylaxis in FPC (Brault et al., 2019; McAtee et al., 2026). Future work is required to understand the impact of injectable metaphylaxis on the microbiome of the respiratory tract in FPC.

*H. somni* was detected in more YRL at 14 DOF than at arrival using metagenomic sequencing and trended similarly for FPC, aligning with a previous report from western Canadian feedlots comparing prevalence at arrival to 90 to 120 DOF (Erickson et al., 2017). In our previous work, the odds of detection were higher for culture or metagenomics at 13 DOF vs. arrival in FPC that received oxytetracycline metaphylaxis, but detection in FPC receiving tulathromycin metaphylaxis varied by year (Abi Younes et al., 2024, 2025).

Detection of *M. bovis* was consistently higher at 14 DOF than arrival with all tests on FPCs, but only with metagenomics in YRL. This was interesting given that the sensitivity for detection was lower with metagenomics compared to qPCR or RPA. However, the higher detection after arrival in FPC was expected (Castillo-Alcala et al., 2012; Becker et al., 2020; Catania et al., 2020). Risk factors for *M. bovis* relate to the stress of transport and co-mingling of calves, with subsequent transmission upon arrival at the feedlot (Caswell and Archambault, 2007; Maunsell et al., 2011).

The age and temporal comparisons of AMR detection in *M. haemolytica, P. multocida*, or *H. somni* showed some consistent patterns between multiple tests. The results of AST and metagenomics agreed sporadically, whereas RPA results more often yielded different and sometimes contradictory results. The odds of detection of macrolide resistance by metagenomics and AST in *Pasteurellaceae* were higher at 14 DOF vs. arrival in FPC and higher in FPC compared to YRL at 14 DOF. This could be linked to the use of macrolides for metaphylaxis at arrival in the FPC age group (Brault et al., 2019; McAtee et al., 2026) and the subsequent development of resistance after this exposure (Abi Younes et al., 2022; Sarchet et al., 2022; Abi Younes et al., 2024, 2025; Rattanapanadda et al., 2025). Results were similar for tetracycline resistance identified in *Pasteurellaceae*, which was also higher in FPC at 14 DOF vs. arrival for both AST and metagenomics. Interestingly, metagenomic detection of *tet(H)* on target bacterial reads was higher in FPC than YRL and at 14 DOF compared to arrival in both age groups. Detection of the ICE-associated target variants with *tet(H)* by metagenomics in these bacterial reads mirrored *tet(H)*, but this was not echoed by RPA. The 14 DOF vs. arrival relationships in FPC for macrolide and tetracycline resistance are consistent with our previous work that assessed AMR using both AST (Abi Younes et al., 2024) and long-read metagenomics (Abi Younes et al., 2025).

Detection of macrolide and tetracycline resistance by RPA was inconsistent and at times discordant with metagenomics and AST. For tetracyclines, this likely reflects the narrow target of the RPA assay, which detects only ICE-associated *tet(H)* variants (Funk et al., 2025), thereby missing *tet(H)* genes not linked to ICE-associated targets. Additionally, similar *tet(H)*-transposase configurations may be present in non-target *Pasteurellaceae* species (Eidam et al., 2015; Cameron et al., 2019), further complicating interpretation.

Inconsistencies were also observed for macrolide resistance detected by RPA, particularly the lower detection in FPC at 14 DOF compared to arrival. Unlike AST, which evaluates resistance in culture-confirmed isolates, and metagenomics, which restricted ARG detection to reads classified as BRD bacteria, the RPA assay for *msrE-mphE* and *erm(42)* detects ARGs irrespective of host species (Funk et al., 2025). Metagenomic analysis of discordant samples identified *msrE-mphE* in *Klebsiella pneumoniae* and *E. coli*, indicating that sole reliance on RPA could lead to different pen-level antimicrobial treatment decisions than AST or metagenomics.

To date, this and our previous work are the only investigations directly comparing culture and AST with long-read metagenomic sequencing and RPA for detection of BRD bacteria and AMR. Unlike earlier work conducted on commercial FPC but under more controlled research feedlot conditions (Abi Younes et al., 2024, 2025), the current study used samples collected by feedlot veterinary clinics from multiple large, geographically distributed, commercial feedlots in western Canada. The previous study included cohorts of FPCs in two separate years (2020 + 2021) and only compared culture and AST to long-read metagenomics, with no reporting of *M. haemolytica* serotypes or *M. bovis*. The RPA testing for 2021 was reported separately (Funk et al., 2026).

Based on the BCLM analysis, all tests demonstrated moderate to low sensitivities for the detection of *M. haemolytica, P. multocida*, or *H. somni*, while specificities were better. The relative performance between tests for each BRD bacterial species was variable. Estimates for the sensitivities of AST for the detection of macrolide AMR at the sample level in any of *M. haemolytica, P. multocida*, or *H. somni* were consistently higher than that of long-read metagenomics or RPA regardless of the combinations of AMR examined, although there was room for improvement in sensitivity overall. In particular, the sensitivity of metagenomics was very low for the detection of *estT* compared to AST-determined resistance to 16-membered ring macrolides. While *estT* has been described as associated with resistance to 16-membered ring macrolides (Dhindwal et al., 2023), the low sensitivity of detection of *estT* by metagenomics suggests that its presence alone was not a sufficient indicator of macrolide resistance in these samples.

Specificities of metagenomics for macrolide ARGs were similar to that of AST except for the model where a positive result for metagenomics included any ARG associated with macrolide AMR, where the specificity was lower than AST. Many of the ARGs included in the full list have complex and non-specific AMR mechanisms, which may contribute to the lower specificity compared to phenotypic macrolide resistance and to models with a limited list of ARGs more explicitly associated with macrolide resistance [e.g., *msrE, mphE, erm(42), estT*] in *Pasteurellaceae* organisms. For example, genes identified in our data included genes described in CARD as components or regulators of multidrug efflux complexes (e.g., *mdtE, mdtF, TolC, crp, gadW, gadX*, and *hns*; Alcock et al., 2023). This speaks to a limitation of defining AMR as detection of either phenotypic AMR by AST or ARGs by DNA-based tests. Genomic detection of AMR is also limited to the known ARGs that are characterized and reported in databases but excludes the novel determinants yet to be identified. Additional work to explore functional genomics and *in silico* mapping of ARGs to flanking regulators and other genetic regulatory mechanisms could provide additional insight (Su et al., 2019; Sherry et al., 2025) but have been more commonly applied in whole genome sequencing of pure isolates rather than metagenomics.

While we were able to estimate the sensitivity and specificity of both AST and metagenomics for florfenicol resistance, the prevalence of phenotypic resistance was very low, necessitating cautious interpretation. Similarly, it was not possible to generate estimates for fluoroquinolone or third generation cephalosporin resistance due to low prevalence of resistant phenotypes and ARG detection, which limited the power to evaluate these tests for resistance associated with these other antimicrobials used to treat BRD (Dowling, 2025; Dowling and Lardé, 2025; Hardefeldt and Prescott, 2025).

Estimates of sensitivity in all AMR models were limited by the intentional strategy of requiring detection of one of *M. haemolytica, P. multocida*, and/or *H. somni* followed by detection of AMR or ARGs. The moderate sensitivity for detection of each organism thus necessarily leads to a limited sensitivity for detection of associated AMR and ARGs. Testing a single isolate per species also potentially limited the sensitivity of AST for characterizing the sample, given that susceptibility can potentially vary across isolates of the same species within a single sample (Capik et al., 2017; Carter et al., 2022). However, the intent of this study was to describe performance for tests that would be available and used by practitioners in commercial feedlot cattle raised under field conditions. This study employed the culture and AST procedure used by most commercial laboratories for both diagnostic and surveillance samples. While detection of ARGs using metagenomics was not limited to a single isolate as it would be for whole genome sequencing, ARGs were reported when their sequences were identified on reads classified as bacteria of interest. This contrasts most metagenomic protocols that identify organisms and ARGs within a sample, but without the ability to assert the linkage of specific ARGs within the taxonomic origin of the read (Taxt et al., 2020; Whittle et al., 2022; Zhang et al., 2022; Ahmadi et al., 2023; Chen et al., 2023; Liu et al., 2023; Ring et al., 2023; Herman et al., 2024).

The specificity of RPA for the detection of macrolide AMR was consistently lower than that of metagenomics or AST. Testing for *msrE-mphE* and *erm(42)* by RPA was limited to samples that had tested positive for one of the target bacteria as a strategy to approximate testing for ARG linked to these bacteria (Funk et al., 2025). However, the RPA assay for *msrE-mphE* and *erm(42)* can detect these ARGs within other bacterial species in these samples, such as *Klebsiella pneumoniae* and *E. coli*. This finding may explain, at least in part, the lower specificity for RPA than metagenomics or AST for the detection of resistance determinants linked to *M. haemolytica, P. multocida*, or *H. somni*.

Overall, results for detection of organisms were similar to our previous, large-scale metagenomic study in a research feedlot (Abi Younes et al., 2025). There was a trend toward lower sensitivities for culture and metagenomics for samples from the present field study. While most differences were not significant, some estimates were significantly lower than previously reported including for *M. haemolytica* detection by culture or metagenomics and for *P. multocida* or *H. somni* detection by metagenomics. Similarly, the sensitivities of detection of both macrolide and tetracycline AMR by AST or metagenomics were also generally lower in the current field study, while specificities were similar (Abi Younes et al., 2025). Field samples shipped to the lab in this study were subject to longer transit times with subsequent potential for impact from temperature fluctuations compared to the samples from our previous study that were transported to the lab immediately. Transit time could impact the microbial composition for culture, prior to enrichment for metagenomics, and DNA quality for RPA testing. Exclusion of samples with transit times longer than 2 days had minimal impact on the BLCM results for selected models, suggesting our methods were relatively robust to shipping delays provided samples were collected and transported to the lab within 48 h. However, the limited number of samples that required more than 48 h to reach the laboratory reduced the ability to detect differences. Future work to understand the impact that transit time has on our ability to interpret these data are warranted.

In addition to intrinsic test performance (diagnostic sensitivity and specificity), clinicians must be able to evaluate the probability that the positive and negative results of a test represent the true status of BRD bacteria and antimicrobial susceptibility in that animal or group of animals, namely the PPV and NPV (Dohoo et al., 2009). Our previous work estimated PPV and NPV from metagenomics but did not consider the important macrolide ARGs detected by metagenomics in combination [*msrE-mphE, erm(42)*, and/or *estT*] and did not make a comparison to phenotypic macrolide resistance (Abi Younes et al., 2025). The PPV and NPV for metagenomic detection of *tet(H)* were somewhat different for this field study, largely owing to the higher estimated sensitivity for detection by metagenomics in the previous study at a research feedlot (Abi Younes et al., 2025).

The relative importance of PPV vs. NPV for detection of macrolide or tetracycline AMR in BRD bacteria depends on the context and overarching goal of testing. Our metagenomics and AST tests had good specificities and subsequent PPVs compared to RPA; all three tests suffered from lower sensitivity and subsequently lower NPVs. A high PPV is often desired when the costs (e.g., finances, time, and personnel) associated with a positive test result are high (Trevethan, 2017). In a regulatory environment where diagnostic evidence is required to justify the use of an antimicrobial for disease management in food animals as a strategy to mitigate AMR, a higher PPV may be preferred to provide high confidence in positive test results that then necessitates a change in antimicrobial protocol. From a clinical decision-making perspective, clinicians may put more weight on a high NPV if the costs of false negatives are higher, and where timely and effective treatment can limit further transmission (Trevethan, 2017).

The balance between PPV and NPV depends on the underlying prevalence. The high specificities of both metagenomics and AST for macrolides and tetracyclines mean that the PPV is reasonable when the prevalence of AMR is >20–30% but falls when the prevalence dips below 20%. Given the low sensitivity of all three tests, our study demonstrated that the NPV of any of the three tests for macrolide or tetracycline AMR would be high when the prevalence is less than 20% but tapers dramatically above 20%. To implement a testing strategy predicated on providing pen- and feedlot-level estimates of AMR in BRD bacteria to inform antimicrobial decisions about future animals that get sick (Otto et al., 2024), veterinary practitioners would need to identify actionable thresholds above which they would make decisions to change antimicrobial treatment protocols for BRD under a testing-informed treatment model. Based on current AMR surveillance (CFAASP, 2022), current antimicrobial therapy for BRD is effective from the perspective of low-levels of phenotypic AMR. Utilizing existing data, agent-based modeling of BRD AMR in western Canadian commercial feedlots identified that antimicrobial interventions in response to BRD would only benefit from testing-informed treatment if AMR reached extreme levels (Ramsay D. E. et al., 2025). Interviews of Canadian feedlot practitioners identified that testing-informed treatment strategies must have demonstrable health benefits to be adopted as mainstream practice (Adewusi et al., 2024), which would require this determination of actionable thresholds.

This study was unique in examining ICE-associated target variants by both metagenomics and RPA as well as the application of BLCMs to compare detection of *M. haemolytica* serotypes. Specificity for the detection of ICE-associated target variants was lower for RPA than metagenomics; sensitivity was not different. The metagenomic assay identified ICE-associated target variants on individual reads classified as the target BRD organisms whereas RPA has the potential to identify ICE-associated target genes that may not be on the target BRD organisms. The performance of metagenomics and RPA for detection of genomic regions associated with serotypes A1 and A6 was very similar, with high specificity but relative low sensitivity estimates for both tests. As a result, the NPVs for the detection of serotypes A1 and A6 by serotype-specific metagenomics and RPA were poor compared to culture and metagenomics for detection of any *M. haemolytica* serotype. Conversely, the PPVs were all similar and greater than 75% if the prevalence was above 30%.

In the model comparing metagenomics and RPA to qPCR for the detection of *M. bovis*, a cutoff of at least 2 reads was necessary to obtain a specificity of 0.90 for metagenomics. This resulted in a sensitivity that was significantly lower than both RPA and qPCR. It is possible that compared to the other BRD bacteria of interest in this study, the non-selective enrichment step may have favored the growth of the other three organisms and reduced the relative presence of *M. bovis* in these samples. However, we were still able to detect *M. bovis* using long-read metagenomics despite this enrichment, which was necessary to reliably detect ARGs. Previous work without this additional step identified *M. bovis* slightly more frequently but did not consistently identify ARGs (Donbraye et al., 2026a,b).

Bayesian latent class models present a powerful method to assess diagnostic test performance without the assumption of a gold standard reference, but as with all models, they are subject to assumptions that can affect the validity of estimates. A key assumption is that the tests under consideration are conditionally independent. In the present study, covariance terms were included between tests to account for dependence based on detection of DNA. We also considered dependence between culture and metagenomics due to the non-selective enrichment step. However, this had no significant impacts on the results, and the more parsimonious models with only covariance between metagenomics and RPA were reported.

A second key assumption is that the populations used in the models have distinctly different prevalence levels. The population groups were chosen because the prevalence levels of BRD bacteria were expected to differ in FPC compared to YRL (Rattanapanadda et al., 2025) and also between the time of arrival at a feedlot and 14 DOF due to mixing and transmission (Abi Younes et al., 2024, 2025; Ramsay D. et al., 2025; Ramsay D. E. et al., 2025). For many of the comparisons between age groups and sample timing, differences were statistically significant when assessed in the present study, indicating that these populations had different prevalences of organism detection and AMR. A third key assumption is that test performance is constant across populations (FPC vs. YRL and arrival vs. 14 DOF); serial exclusion of each population from the model for detection of *M. haemolytica* suggested that test performance was consistent for a key organism in this study.

When comparing genotypic and phenotypic AMR results, it is commonly assumed that genotype is highly correlated with phenotype, which is not always the case (Owen et al., 2017; Rebelo et al., 2022). The lower specificity of models that included any macrolide or any tetracycline ARG as an outcome speak to this. There is an increasing push to use genomics for AMR surveillance and detection given the increasingly affordable and rapid nature of the technology, but care must be taken that sequencing and bioinformatic methods are robust and correlated with epidemiologic data (Sherry et al., 2025). It is also critical to recognize that bioinformatic analyses summarize and report findings that only consider previously characterized and reported traits (e.g., ARGs) and interpretation may miss novel features that are present in the genomics data but have not yet been reported. This study demonstrates the utility of long-read sequencing of samples from a large, geographically distributed study of feedlot cattle and the ability to detect ARGs linked to specific BRD bacteria without the need for bioinformatic assembly of the data (Herman et al., 2024). Future work to understand linkages between ARG detection and phenotypic AMR using functional genomics and *in silico* prediction by machine learning could explore the ability to assess regulatory mechanisms with long-read, metagenomic sequencing data and the ability to better understand how ARGs are expressed (Su et al., 2019; Sherry et al., 2025). Further work to better understand the relationship between AMR and clinical BRD outcomes in feedlot cattle at the individual and pen-level is also required (Andrés-Lasheras et al., 2021; Otto et al., 2024).

## 5 Conclusion

This was the first large field study to use a recently developed long-read metagenomic protocol in a commercial laboratory setting for the detection of BRD bacteria and ARGs in western Canadian feedlot cattle, conducted in collaboration with Canadian BRD surveillance efforts. It was also the first to use BLCMs to compare the performance of long-read metagenomic sequencing and RPA to conventional culture and AST, providing estimates of diagnostic sensitivity, specificity, and predictive values relevant to clinical decision-making. Our findings highlight both the potential and current limitations of emerging molecular diagnostics for detection of BRD pathogens and ARGs for informing AMU in commercial feedlot settings, where diagnostic tools must balance timeliness, accuracy, and interpretability. As livestock production faces increasing expectations for demonstrable antimicrobial stewardship, this integrated testing framework offers a scalable approach for BRD and AMR surveillance that could be adapted to other food animal production systems and resistance monitoring programs.

## Data Availability

The datasets presented in this study can be found in online repositories. The names of the repository/repositories and accession number(s) can be found at: https://github.com/coadunate/ASSETS_2, Sequence Reach Archive (SRA) within BioProject ID: PRJNA1374179.
