# Supplementary material for: Laboratory tests for bovine respiratory bacteria and antimicrobial resistance in commercial feedlot cattle: comparing culture, long-read metagenomics, and recombinase polymerase amplification
Source: Front Microbiol. 2026 May 20;17:1806062. doi: 10.3389/fmicb.2026.1806062 (PMC13229862; doi:10.3389/fmicb.2026.1806062)
Supplement: Supplementary file 1 [file Data_Sheet_1.pdf]

Supplement Material 1: Supporting information. Bayesian latent class model construction methodology.

Bayesian Latent Class Model Detailed Methodology

The first set of BLCMs compared the diagnostic test performance of long-read metagenomic sequencing and RPA to culture for the detection of *M. haemolytica, P. multocida*, and *H. somni*. Theoretical coverage was used to classify samples as positive or negative by metagenomic sequencing. Receiver operating characteristic curves (ROC) comparing theoretical coverage to culture were used to determine a baseline cutoff based on maximizing the combined sensitivity and specificity (Youden's index) of metagenomics (R package pROC, (Robin et al., 2011)).

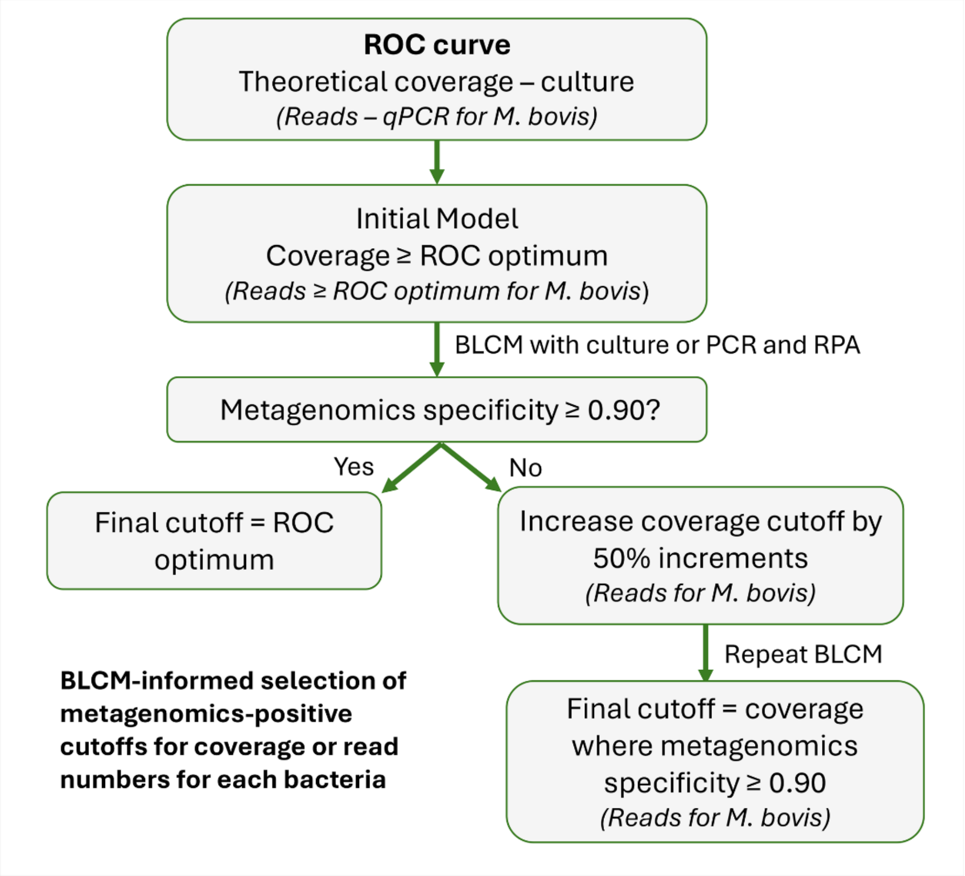

Supplementary Figure S1.1. Development of BLCM-informed thresholds for classification of long-read metagenomic sequencing results for bacteria as positive or negative.
Abbreviations: ROC: receiver operating characteristic; qPCR: quantitative polymerase chain reaction; BLCM: Bayesian latent class model.

The baseline cutoff was used to classify metagenomics for comparison to culture and RPA results for initial BLCMs (results reported in Supplementary Materials 3: Table S3.1). A minimum threshold of BLCM-estimated specificity ≥ 0.90 for metagenomics was selected to ensure clinically acceptable test performance. If the BLCM results using the baseline theoretical coverage cutoff resulted in specificity < 0.90 for metagenomics, the theoretical coverage cutoff was increased by increments of 50% and BLCMs repeated until specificity ≥ 0.90 was obtained for metagenomics. The resultant cutoff was then used to classify long-read metagenomics sequencing as positive or negative for the final models (Supplementary Figure S2.1).

Because a key assumption of BLCMs is conditional independence between tests (Kostoulas et al., 2017; Gardner et al., 2019), the impact of a potential lack of independence between culture and metagenomics due to the bacterial enrichment step was assessed by constructing secondary BLCMs for a subset of the initial models, with additional covariance terms between culture and metagenomics. Results were compared to the primary BLCMs without the extra covariance terms. The RPA test used DNA extracted directly from the swabs and was assumed to be independent from culture.

An additional sensitivity analysis repeated the BLCM analysis for the detection of *M. haemolytica* excluding each of the populations (animal age and sampling time points) in turn to evaluate the impact of potential variation in test performance across populations on the estimates.

A sensitivity analysis was performed to assess whether adjusting coverage for *M. haemolytica* and *P. multocida* for the number of base pairs detected in library preparation water controls impacted BLCM results. This analysis was limited to *M. haemolytica* and *P. multocida* as reads associated with these bacteria were most abundant in control samples. The mean or median read lengths for *M. haemolytica* and *P. multocida* reads detected across control samples for each flow cell were subtracted from the total read lengths for *M. haemolytica* or *P. multocida*, respectively, for each sample from the corresponding flow cell. The adjusted read lengths were then used to calculate mean and median control-adjusted coverage values for each sample. Baseline and final cutoffs for the mean and median control-adjusted data were determined using the same process as for the unadjusted data.

Further BLCMs were developed to compare the clinical sensitivity and specificity for detection of samples with isolates of *M. haemolytica, P. multocida,* or *H. somni* with phenotypic resistance to specific antimicrobials as determined by AST to the detection in samples with known ARGs considered important determinants of corresponding AMR in BRD-associated bacteria by metagenomics or RPA.

The initial model of AMR considered phenotypic resistance to any macrolide (gamithromycin, tulathromycin, tildipirosin, or tilmicosin) by AST, detection of *msrE, mphE, erm(42),* or *estT* genes by long-read metagenomics, and detection of *msrE-mphE* or *erm(42)* genes by RPA. Other specific models included: 1) phenotypic resistance to any macrolide by AST, detection of any gene potentially associated with macrolide resistance by metagenomics, and detection of *msrE-mphE* or *erm(42)* genes by RPA; 2) phenotypic resistance to

gamithromycin or tulathromycin (15-membered ring macrolides) and the detection of *msrE-mphE* or *erm(42)* genes using long-read metagenomics and RPA; 3) phenotypic resistance to tildipirosin or tilmicosin (16-membered ring macrolides) and detection of the *estT* gene using long-read metagenomics (Dhindwal et al., 2023); 4) phenotypic resistance to tetracycline by AST, detection of the *tet(H)* gene using long-read metagenomics, and detection of *tet(H)* as part of the ICE-associated target gene pairs with RPA; and 5) phenotypic resistance to tetracycline, detection of any gene potentially associated with tetracycline resistance using long-read metagenomics, and detection of *tet(H)* as part of the ICE-associated target gene pairs with RPA. Additionally, a model compared detection of florfenicol resistance by AST and the *floR* gene by metagenomics (no RPA assay available).

For AST, a positive test result was defined as the isolation of any of *M. haemolytica*, *P. multocida,* or *H. somni* from DNP swabs collected from individual calves, followed by AST classification of the isolates as resistant to macrolides (in combinations as described above), tetracycline or florfenicol based on CLSI MIC breakpoints. For long-read metagenomic sequencing, a positive result was defined as a sample where there was detection of the ARG of interest on any read identified as *M. haemolytica, P. multocida,* or *H. somni*. The identification of *msrE* and *mphE* was considered in combination and defined as positive if either or both were identified, since these genes are typically arranged in tandem and co-expressed from the same promoter (Desmolaize et al., 2011). For RPA, the assays for *msrE-mphE* and *erm(42)* were only completed if RPA had identified one of the bacteria of interest; if the target bacteria were not identified the sample was considered negative for ARGs as well.

For the primary models, intermediate AST results were classified as susceptible. Models were also run with intermediate AST results classified with resistant (non-susceptible) as a sensitivity analysis of the impact of alternate classification of intermediate AST results.

An additional BLCM assessed the sensitivity and specificity of long-read metagenomics and RPA for detecting the ICE- associated target gene pairs *tnpA-tet(H)* and *ebrB-tet(H).* A sample was considered positive on metagenomics if at least one of the ICE-associated target gene pairs was detected on a read identified as *M. haemolytica*, *P. multocida,* or *H. somni*. For RPA, the ICE assay had only been completed where one of these bacteria had previously been identified in the sample by RPA and the samples were classified as negative for ICE-associated targets if the target bacteria had not been identified.

A BLCM was also developed to compare long-read metagenomics and RPA to qPCR for the detection of *M. bovis*. For this model, the number of reads identified as *M. bovis* were used to classify samples as positive by metagenomics as reads detected were lower than for other bacteria and estimates of theoretical coverage were very low and more challenging to interpret. A baseline of at least one read was used to create an initial BLCM comparing metagenomics to qPCR and RPA, and if the specificity of metagenomics was < 0.90 the number of reads was increased until the BLCM estimated a metagenomics specificity of at least 0.90 (Supplementary Figure S2.1).

The final BLCM model compared detection of serotypes A1 and A6 of *M. haemolytica* by metagenomics and RPA, given that the RPA assay was designed to target only genes associated with *M. haemolytica* serotypes A1 and A6. For this model, detection of genomic regions associated with serotype A1 or A6 by metagenomics on at least one read identified as *M. haemolytica* was considered positive.

A final sensitivity analysis assessed the impact of transit time to the laboratory on the BLCM estimates across a range of model outcomes. For this analysis, models were limited to a subset of samples that arrived at the diagnostic laboratory within 2 days (n=620), and results were compared to those from the full set of samples (n=760).

A summary of sensitivity analyses and the models to which they were applied is provided in Supplementary Figure S2.2.

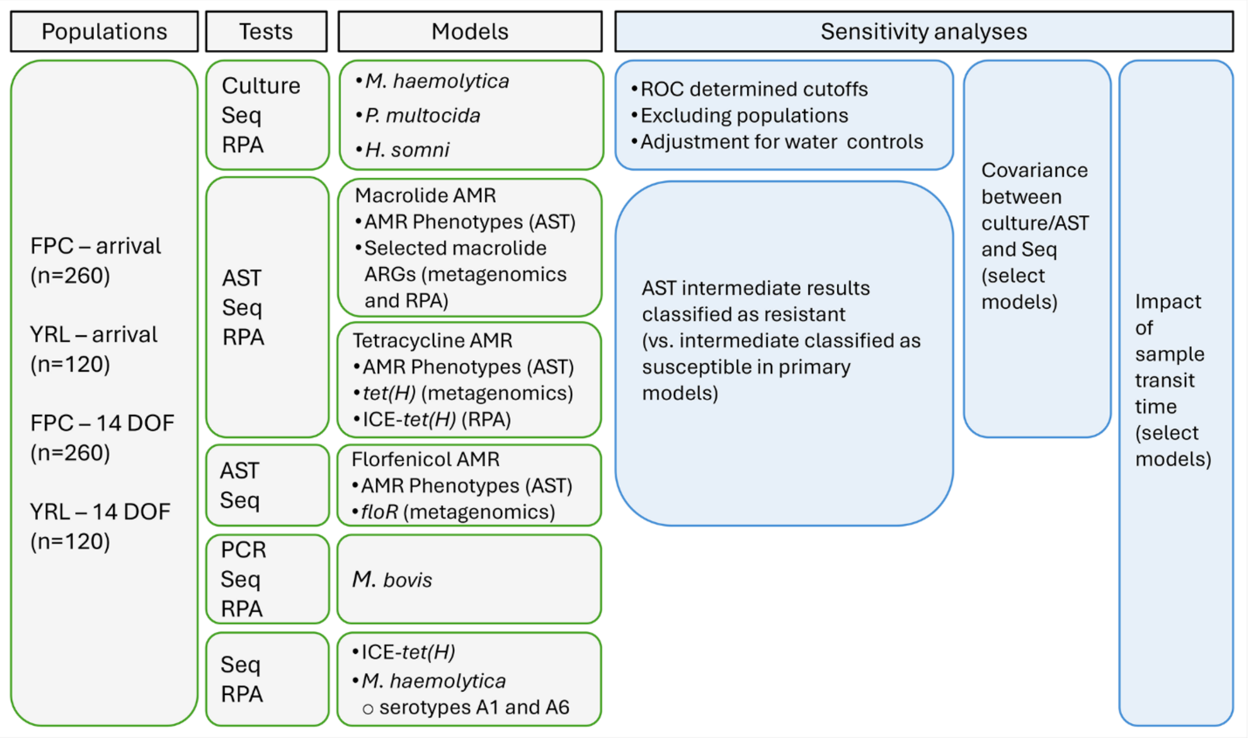

Supplementary Figure S1.2. Summary of sensitivity analyses applied to various BLCM models.

Models compared combinations of tests and outcomes from deep nasopharyngeal swabs collected across four populations of feedlot cattle. Abbreviations: FPC: fall placed calves; YRL: yearlings; DOF: days on feed; Seq: long-read metagenomic sequencing; RPA: recombinase polymerase amplification; AST: antimicrobial susceptibility testing; PCR: polymerase Chain Reaction; AMR: antimicrobial resistance; ARG: antimicrobial resistance gene; ICE: integrative and conjugative element-associated gene targets; ROC: receiver operating characteristic curve.

References

Desmolaize, B., Rose, S., Wilhelm, C., Warrass, R., and Douthwaite, S. (2011). Combinations of macrolide resistance determinants in field isolates of Mannheimia haemolytica and Pasteurella multocida. *Antimicrob Agents Chemother* 55(9)**,** 4128-4133. doi: 10.1128/aac.00450-11.

Dhindwal, P., Thompson, C., Kos, D., Planedin, K., Jain, R., Jelinski, M., et al. (2023). A neglected and emerging antimicrobial resistance gene encodes for a serine-dependent macrolide esterase. *Proc Natl Acad Sci U S A* 120(8)**,** e2219827120. doi: 10.1073/pnas.2219827120.

Gardner, I.A., Colling, A., and Greiner, M. (2019). Design, statistical analysis and reporting standards for test accuracy studies for infectious diseases in animals: Progress, challenges and recommendations. *Prev Vet Med* 162**,** 46-55. doi: 10.1016/j.prevetmed.2018.10.023.

Kostoulas, P., Nielsen, S.S., Branscum, A.J., Johnson, W.O., Dendukuri, N., Dhand, N.K., et al. (2017). STARD-BLCM: Standards for the Reporting of Diagnostic accuracy studies that use Bayesian Latent Class Models. *Prev Vet Med* 138**,** 37-47. doi: 10.1016/j.prevetmed.2017.01.006.

Robin, X., Turck, N., Hainard, A., Tiberti, N., Lisacek, F., Sanchez, J.C., et al. (2011). pROC: an open-source package for R and S+ to analyze and compare ROC curves. *BMC Bioinformatics* 12**,** 77. doi: 10.1186/1471-2105-12-77.
