# Supplementary material for: Laboratory tests for bovine respiratory bacteria and antimicrobial resistance in commercial feedlot cattle: comparing culture, long-read metagenomics, and recombinase polymerase amplification
Source: Front Microbiol. 2026 May 20;17:1806062. doi: 10.3389/fmicb.2026.1806062 (PMC13229862; doi:10.3389/fmicb.2026.1806062)

Supplementary Material 2: Antimicrobial susceptibility testing results from deep nasopharyngeal swabs collected as part of the Canadian Feedlot AMR/AMU Surveillance Program (CFAASP) for bovine respiratory disease bacteria.

**All figures prepared by CFAASP

METHODS

Laboratory data were exported from a central repository to Stata/IC 14.2 for statistical analyses. Minimum inhibitory concentrations (MICs) were interpreted with reference to the breakpoints established by the Clinical Laboratory Standards Institute (CLSI). Isolates with intermediate MIC values were categorized as "susceptible" for all analyses.

Population-averaged prevalences of resistance to single antimicrobial drugs and exact 95% confidence intervals were estimated using null (i.e., intercept-only) generalized estimating equation (GEE) models to account for the clustering of isolate- and animal-level AMR within feedlots. All statistical models included a binary outcome, logit-link function and exchangeable correlation structure. When the prevalence of resistance was 0%, a confidence interval calculator was used to estimate an exact upper confidence interval only.

Statistical outputs were exported to Microsoft Excel and R, and the figures were generated with these applications.

Isolate-level results (Supplementary Figures S2.1-S2.9): percentages calculated using the number of positive isolates for that organism as the denominator.

Sample-level results (Supplementary Figures S2.10-S2.14): percentages calculated using the total number of samples of the specified calf age group and time point as the denominator.

Supplementary Figures S2.4-S2.9 are UpSet plots of antimicrobial susceptibility data.

An UpSet plot allows for visualization relationships between intersecting datasets. Each figure contains the number of isolates across antimicrobial susceptibility patterns and for two time points (arrival [left] and rehandling [14 days on feed – right]). The vertical bar graph at the top shows the number of isolates in each resistance pattern indicated in the dot plot below. The horizontal bar graph to the left of the dot plot shows the total number of isolates corresponding to that specific drug resistance outcome in each row of the dot plot. The dot plot shows the different resistance patterns in vertical columns by antimicrobial drug. Drugs within the same antimicrobial class are given the same colour.

ISOLATE-LEVEL RESULTS

Supplementary Figure S2.1. Percentage of isolates with key resistance phenotypes, select respiratory bacteria by time of sample (*ASSETS* isolates from 2022).

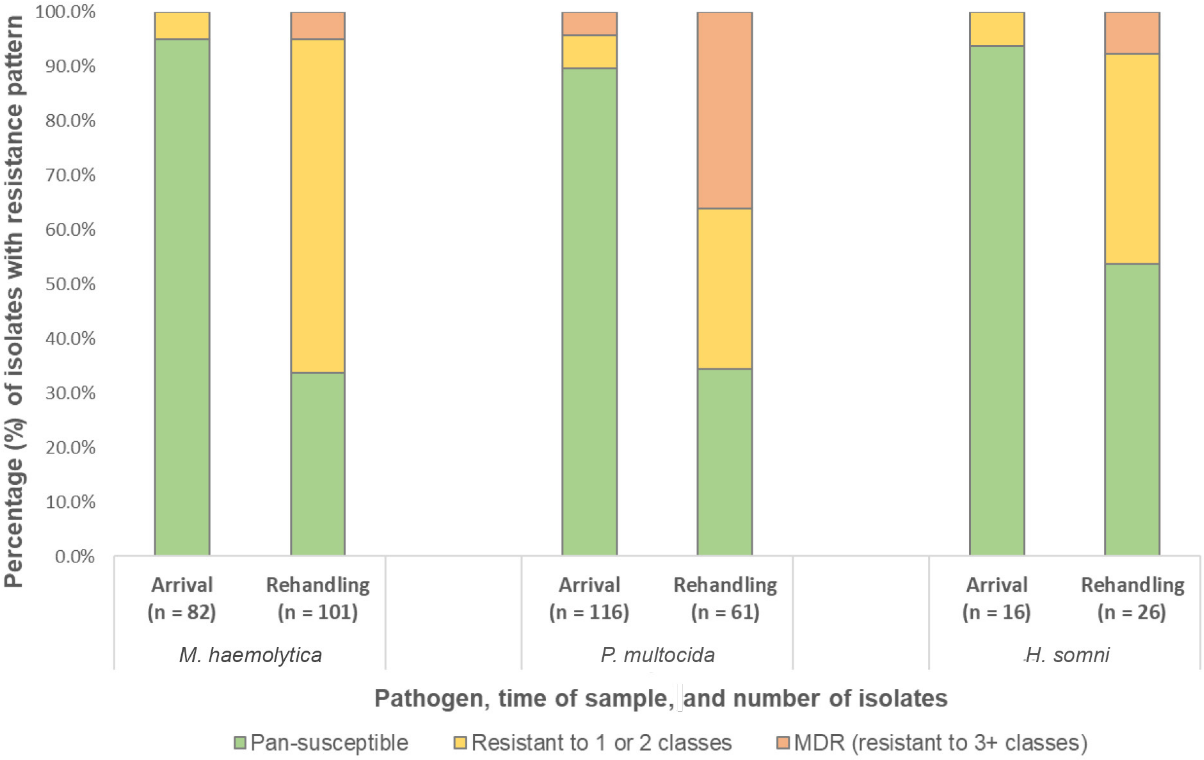

Supplementary Figure S2.2. Percentage of isolates from fall-placed calves (left) and yearlings (right) with key resistance phenotypes, select respiratory bacteria by time of sample (*ASSETS* isolates from 2022).

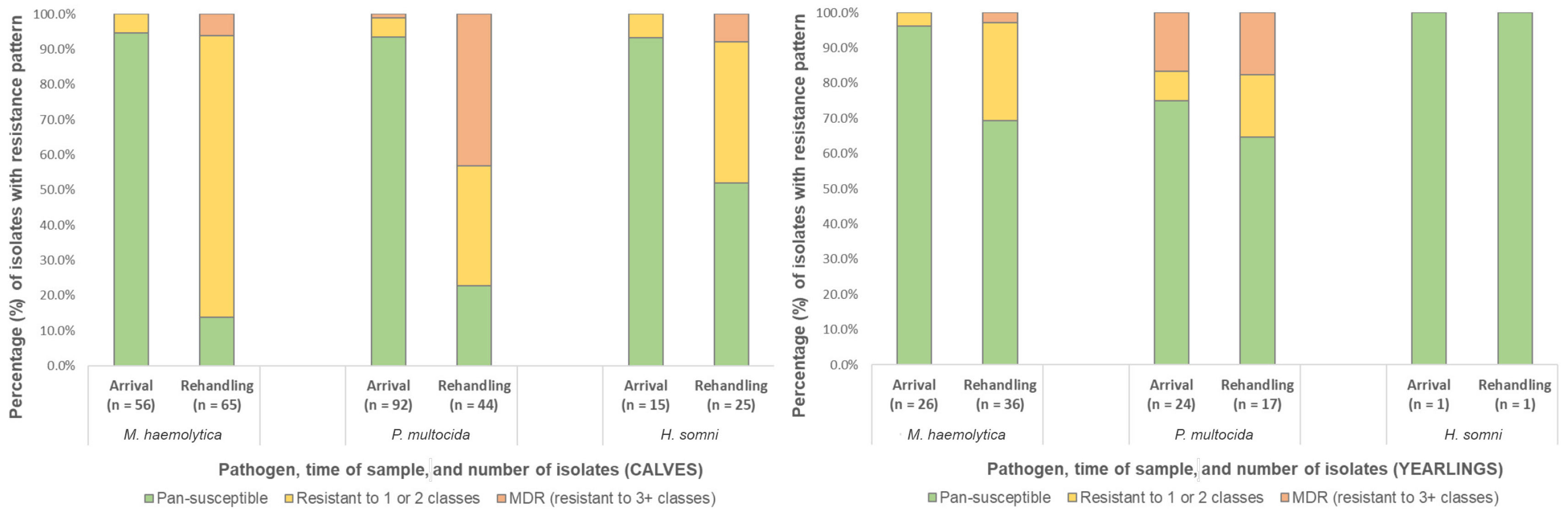

Supplementary Figure S2.3. Percentage of A) *Mannheimia haemolytica,* B) *Pasteurella multocida,* and C) *Histophilus somni* isolates resistant to single antimicrobials at feedlot arrival and at rehandling (*ASSETS* isolates from 2022), adjusted for clustering by feedlot.

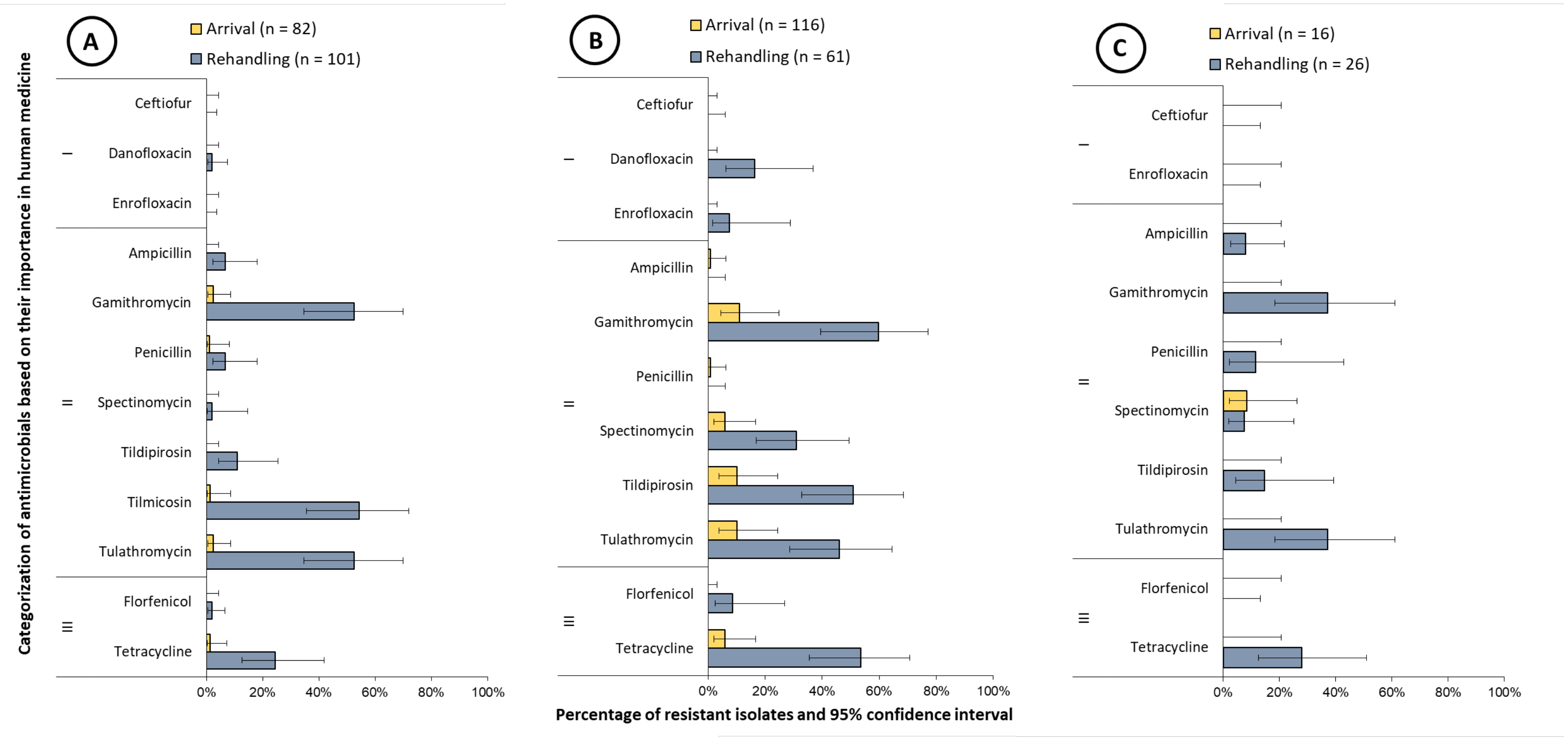

Supplementary Figure S2.4. Resistance profiles at arrival (left, n = 56) and rehandling (right, n = 65) for *M. haemolytica* isolates from calves (*ASSETS* isolates from 2022).

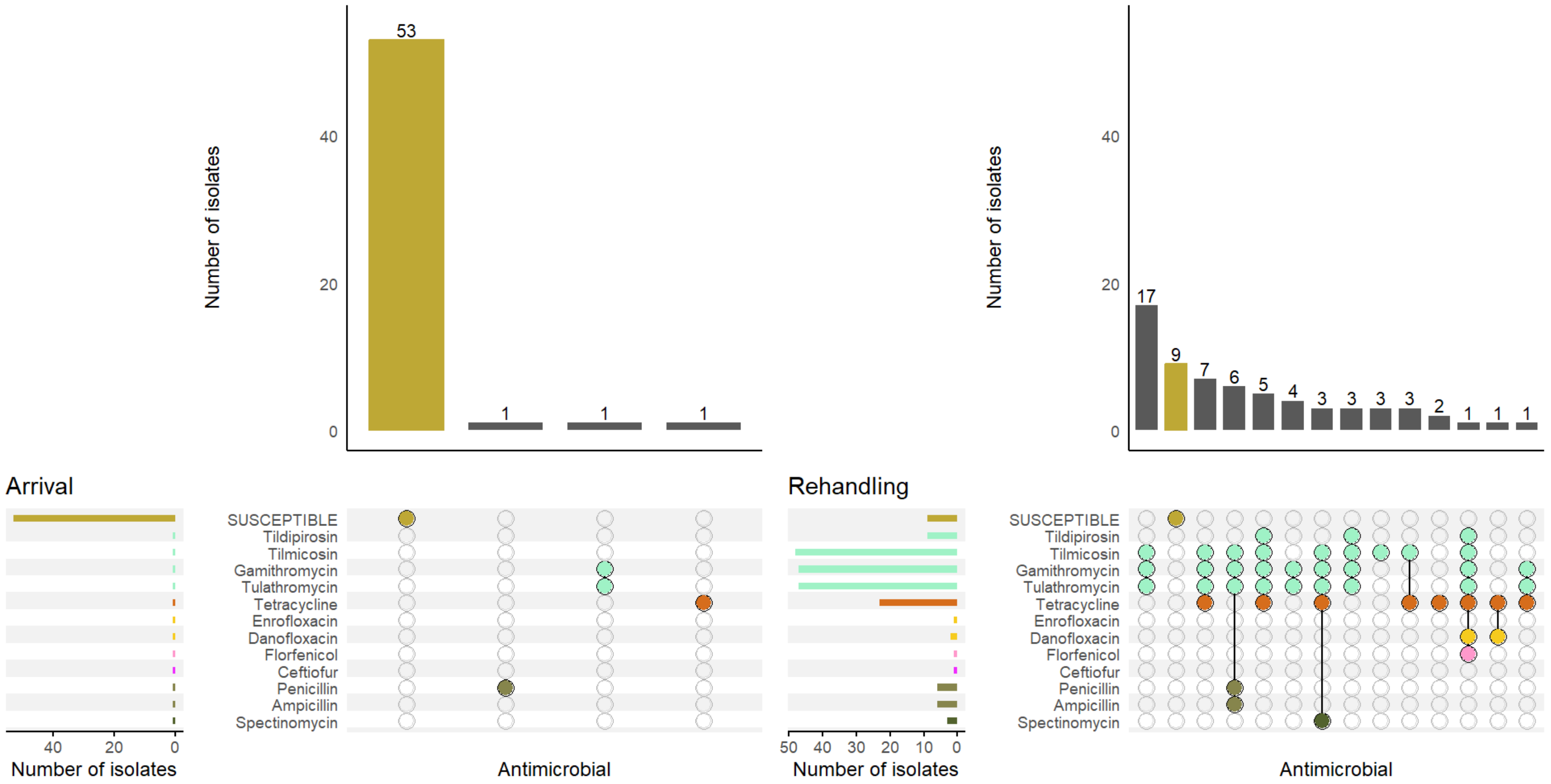

Supplementary Figure S2.5. Resistance profiles at arrival (left, n = 26) and rehandling (right, n = 36) for *M. haemolytica* isolates from yearlings (*ASSETS* isolates from 2022)

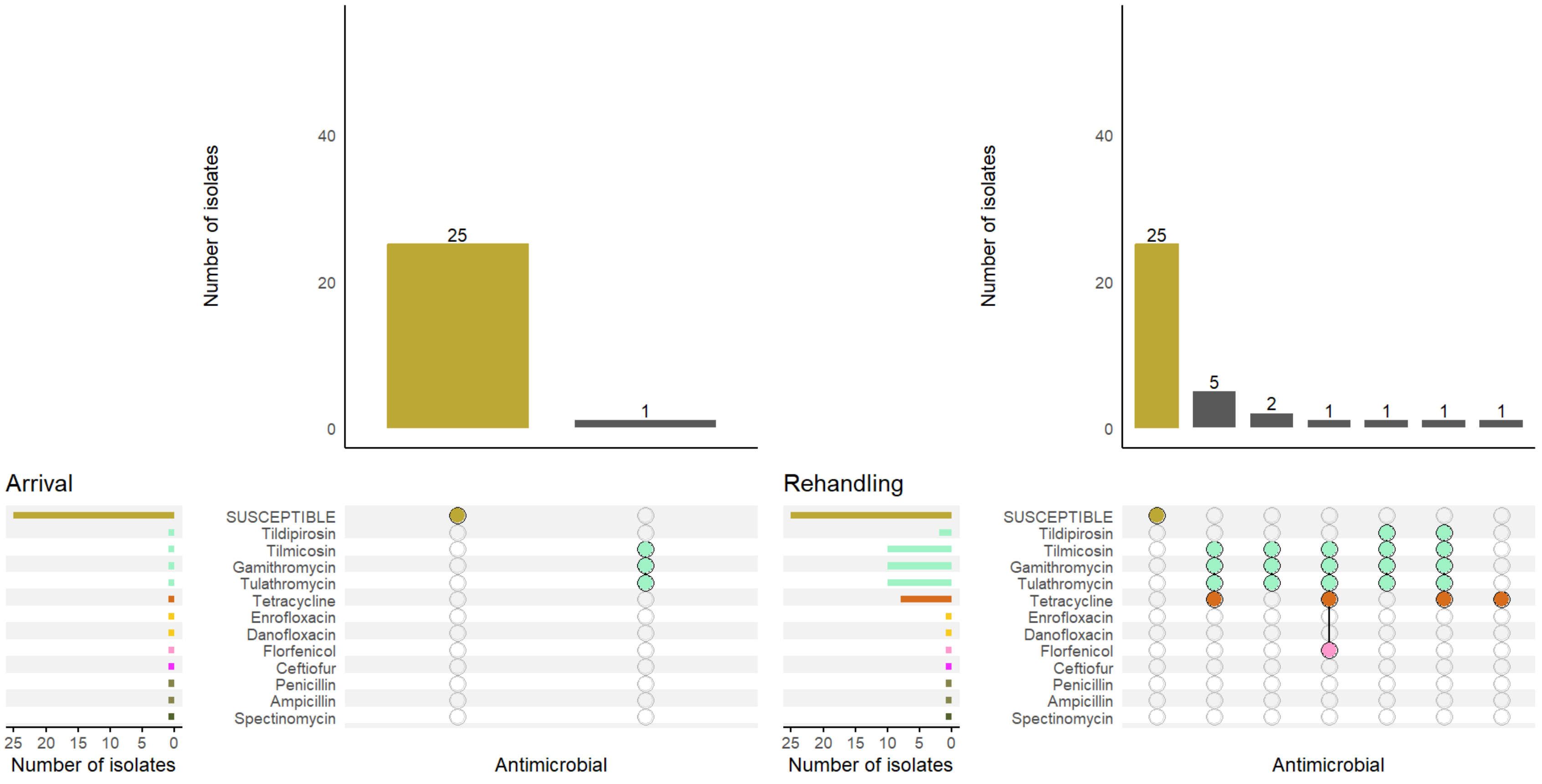

Supplementary Figure S2.6. Resistance profiles at arrival (left, n = 92) and rehandling (right, n = 44) for *P. multocida* isolates from calves (*ASSETS* isolates from 2022)

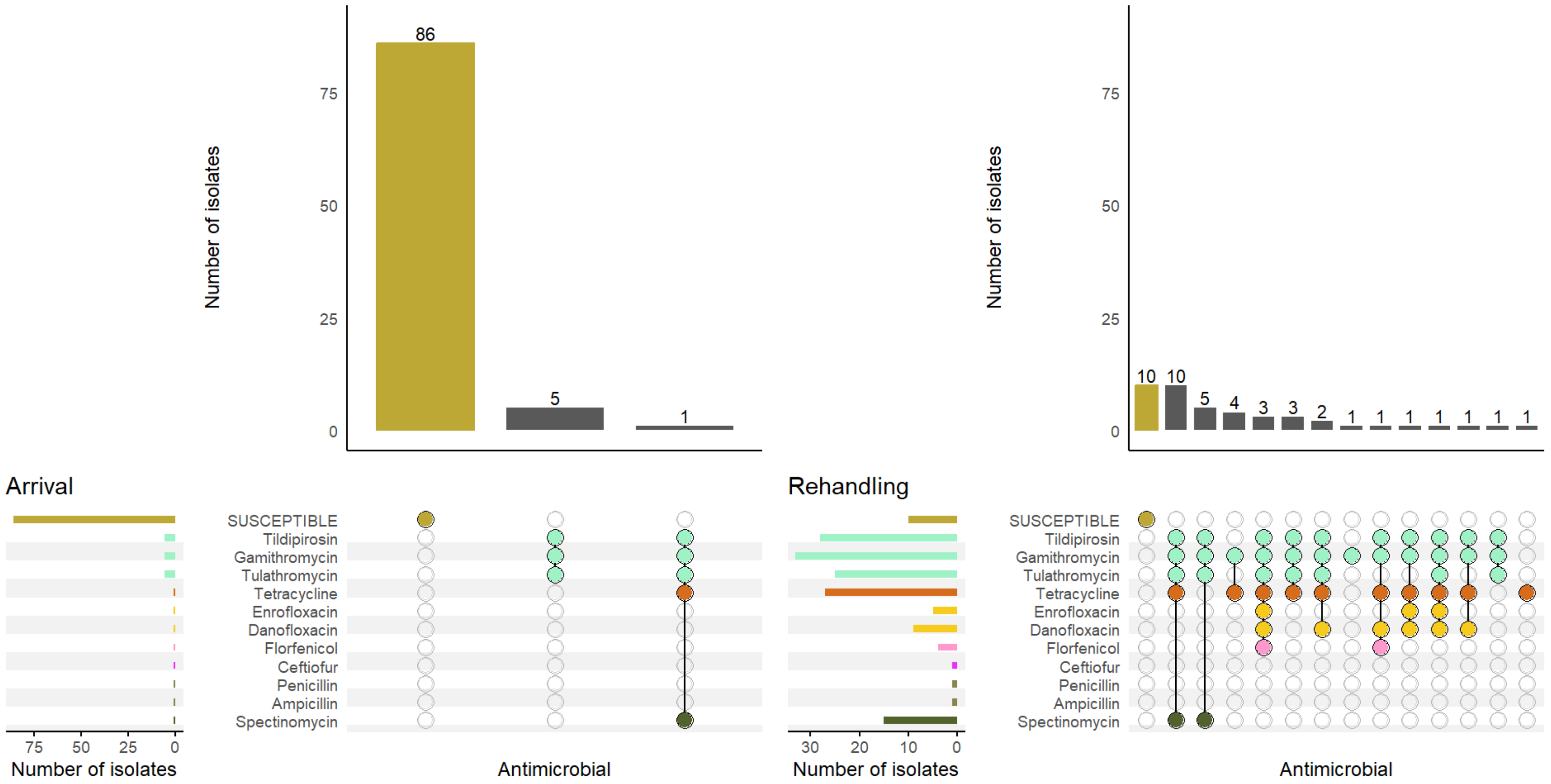

Supplementary Figure S2.7. Resistance profiles at arrival (left, n = 24) and rehandling (right, n = 17) for *P. multocida* isolates from yearlings (*ASSETS* isolates from 2022)

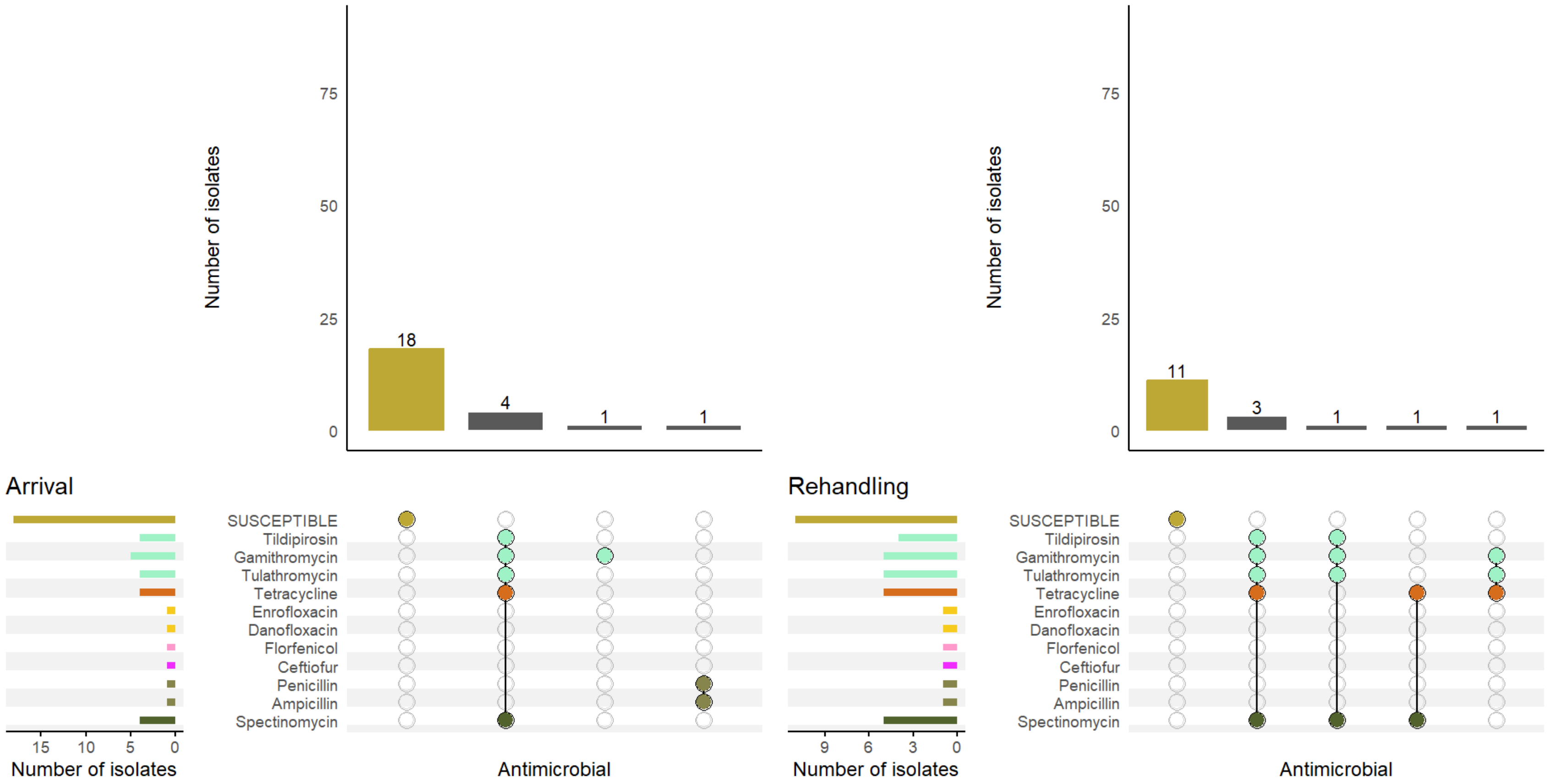

Supplementary Figure S2.8. Resistance profiles at arrival (left, n = 15) and rehandling (right, n = 25) for *H. somni* isolates from calves (*ASSETS* isolates from 2022)

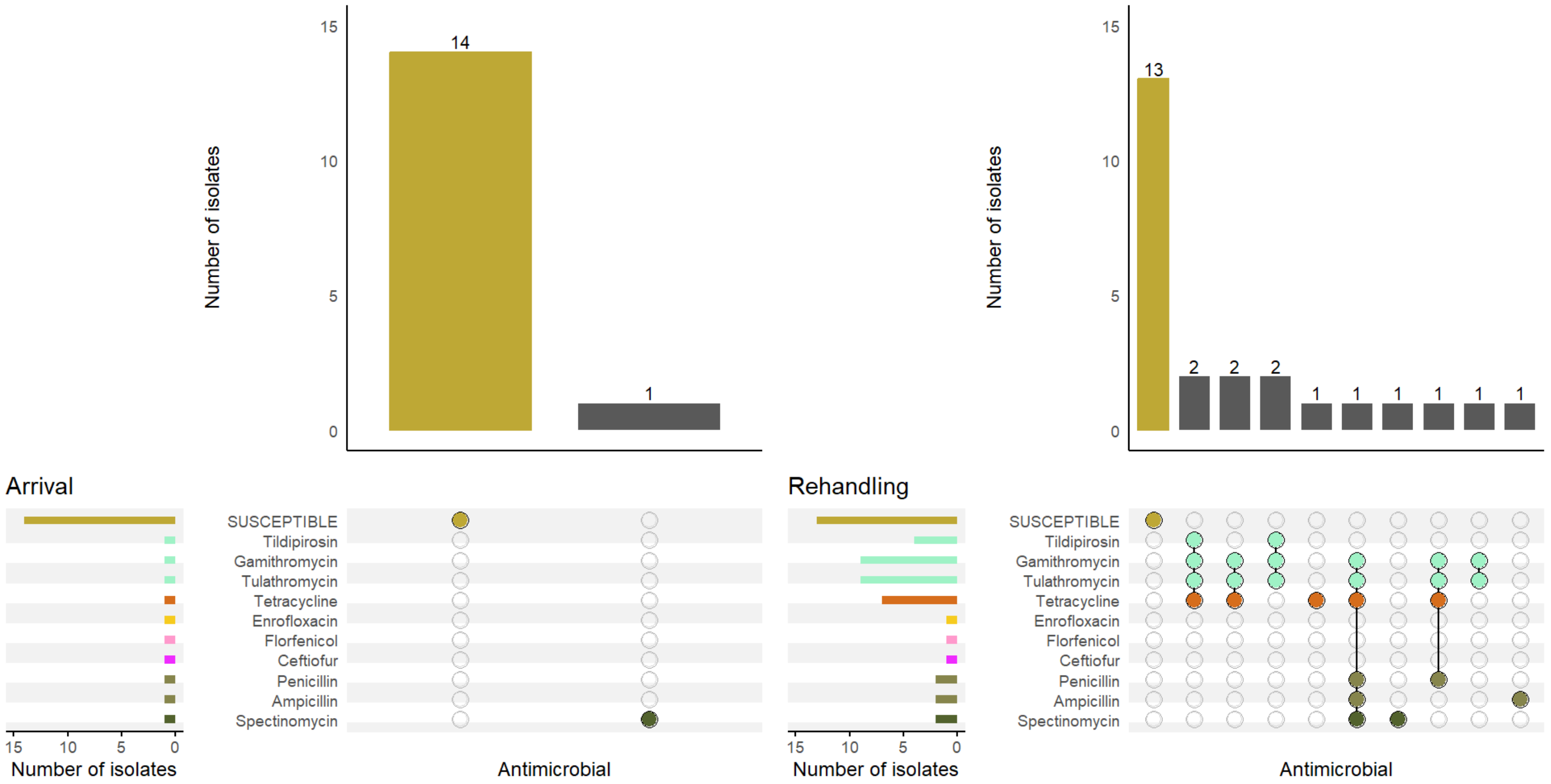

Supplementary Figure S2.9. Resistance profiles at arrival (left, n = 1) and rehandling (right, n = 1) for *H. somni* isolates from yearlings (*ASSETS* isolates from 2022)

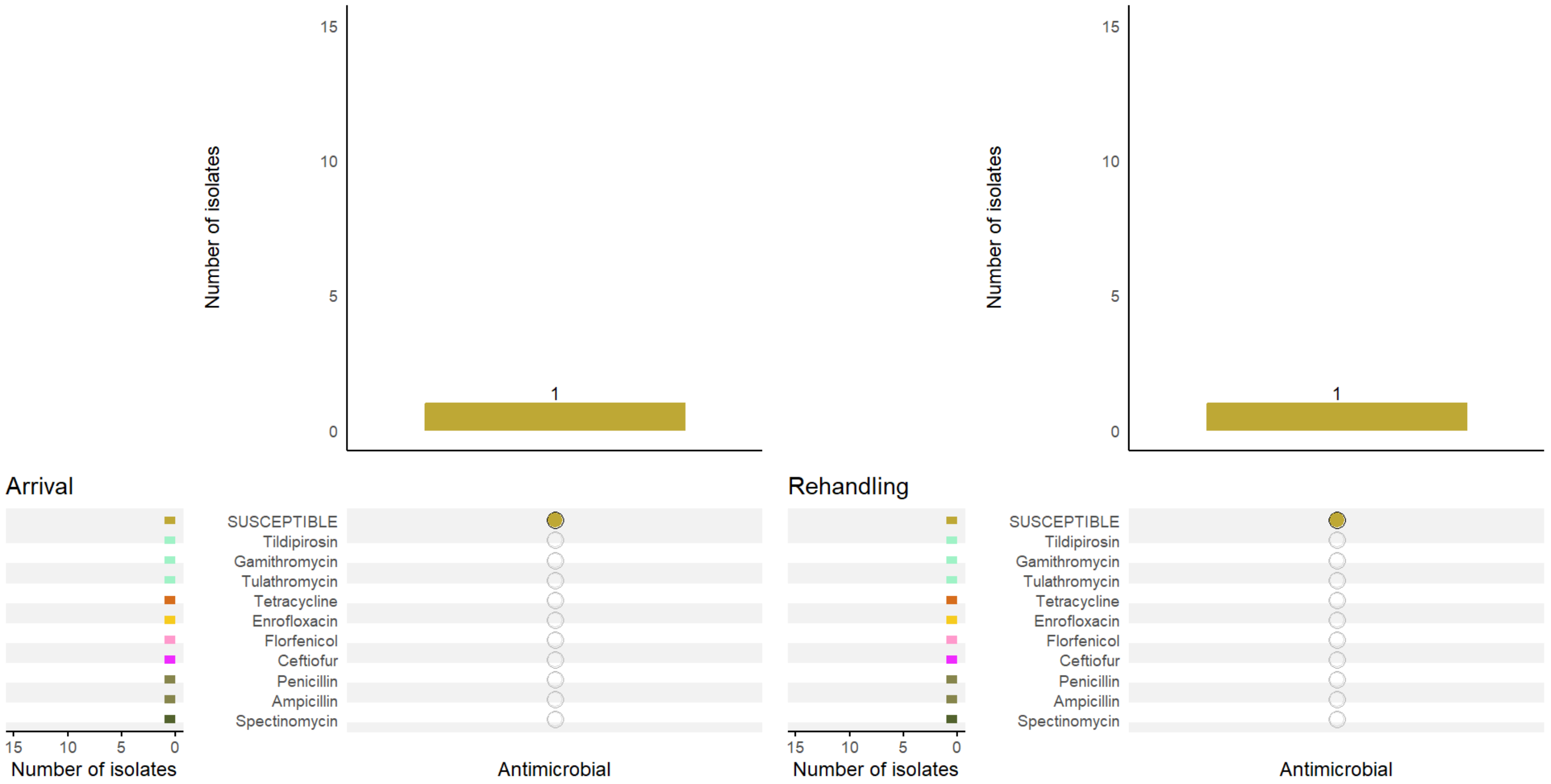

SAMPLE-LEVEL RESULTS

Supplementary Figure S2.10. Percentage of samples with respiratory pathogen-associated resistance phenotype, by time of sample (*ASSETS* samples from 2022)

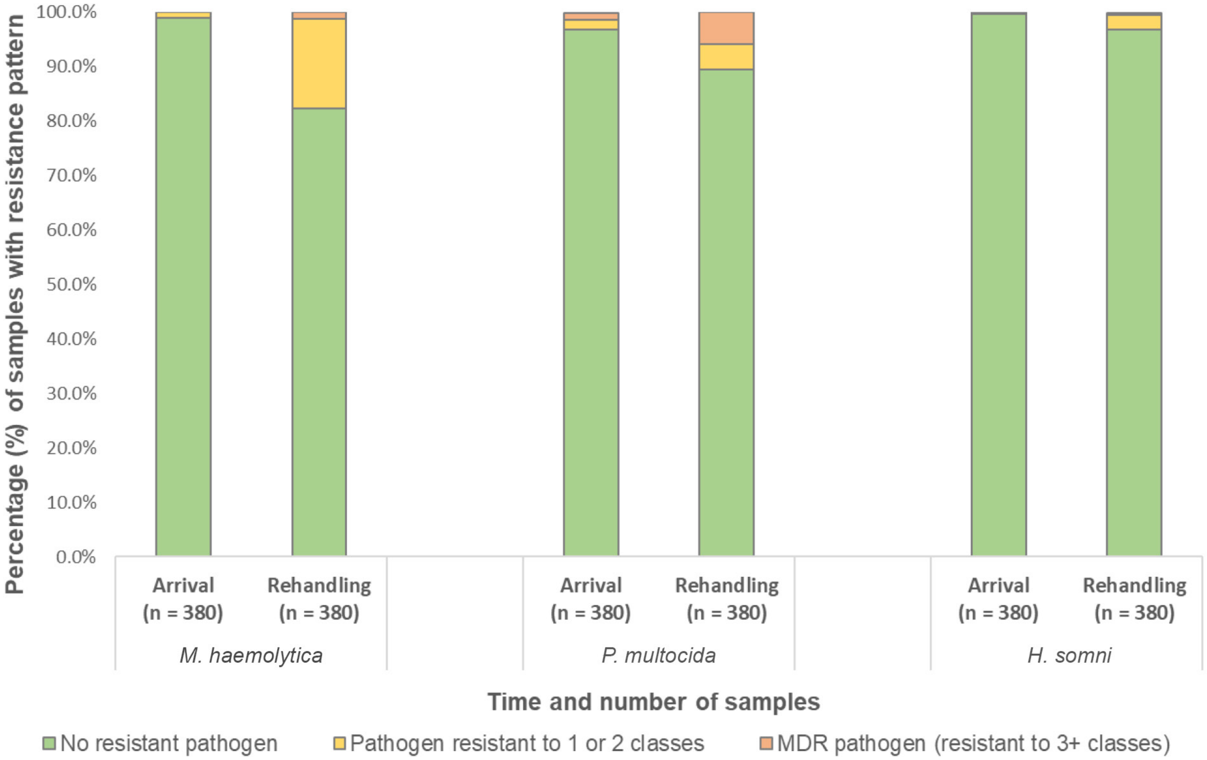

Supplementary Figure S2.11. Percentage of samples from fall-placed calves (left) and yearlings (right) with respiratory pathogen-associated resistance phenotype, by time of sample (*ASSETS* samples from 2022)

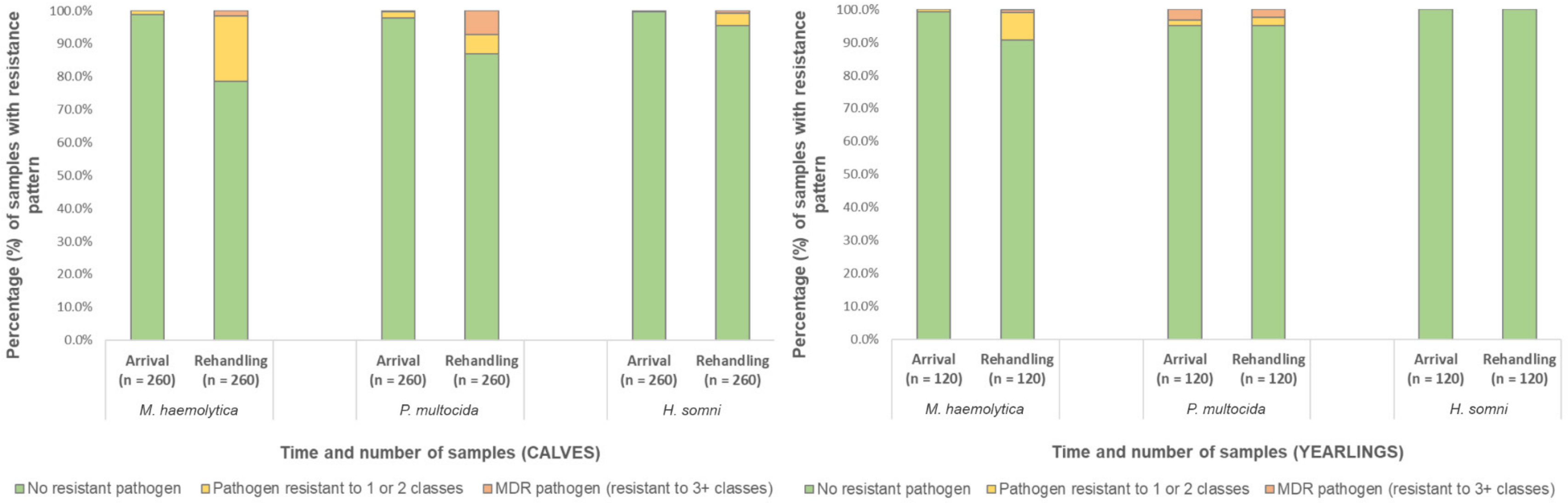

Supplementary Figure S2.12. Percentage of samples with resistance to single antimicrobials in A) *Mannheimia haemolytica*, B) *Pasteurella multocida*, and C) *Histophilus somni* at feedlot arrival and at rehandling (*ASSETS* samples from 2022), adjusted for clustering by feedlot. Note that the maximum boundary for the x-axis is 30% in these figures.

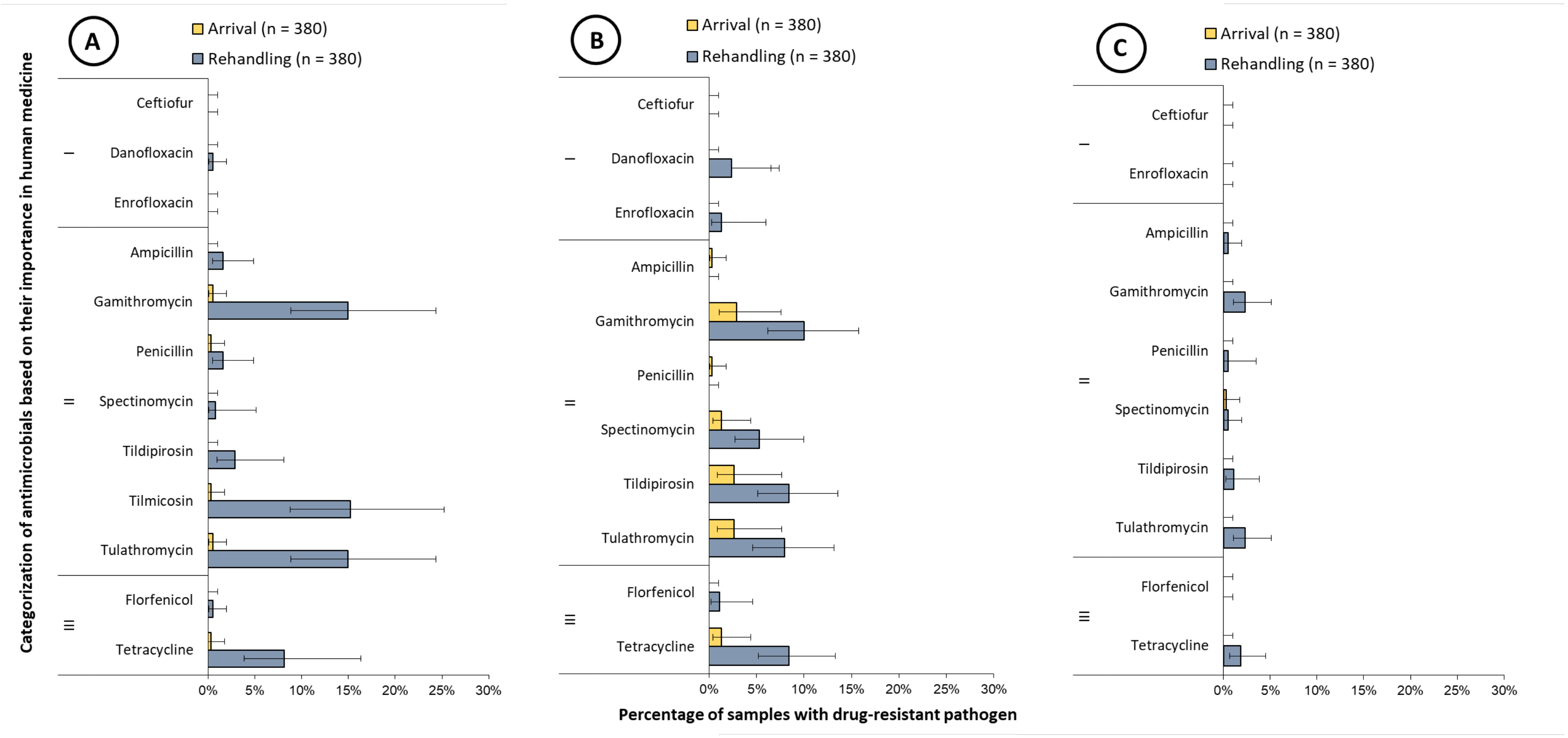

Supplementary Figure S2.13. Percentage of samples from fall placed calves with resistance to single antimicrobials in A) *Mannheimia haemolytica,* B) *Pasteurella multocida,* and C) *Histophilus somni* at feedlot arrival and at rehandling (*ASSETS* samples from 2022), adjusted for clustering by feedlot. Note that the maximum boundary for the x-axis is 30% in these figures.

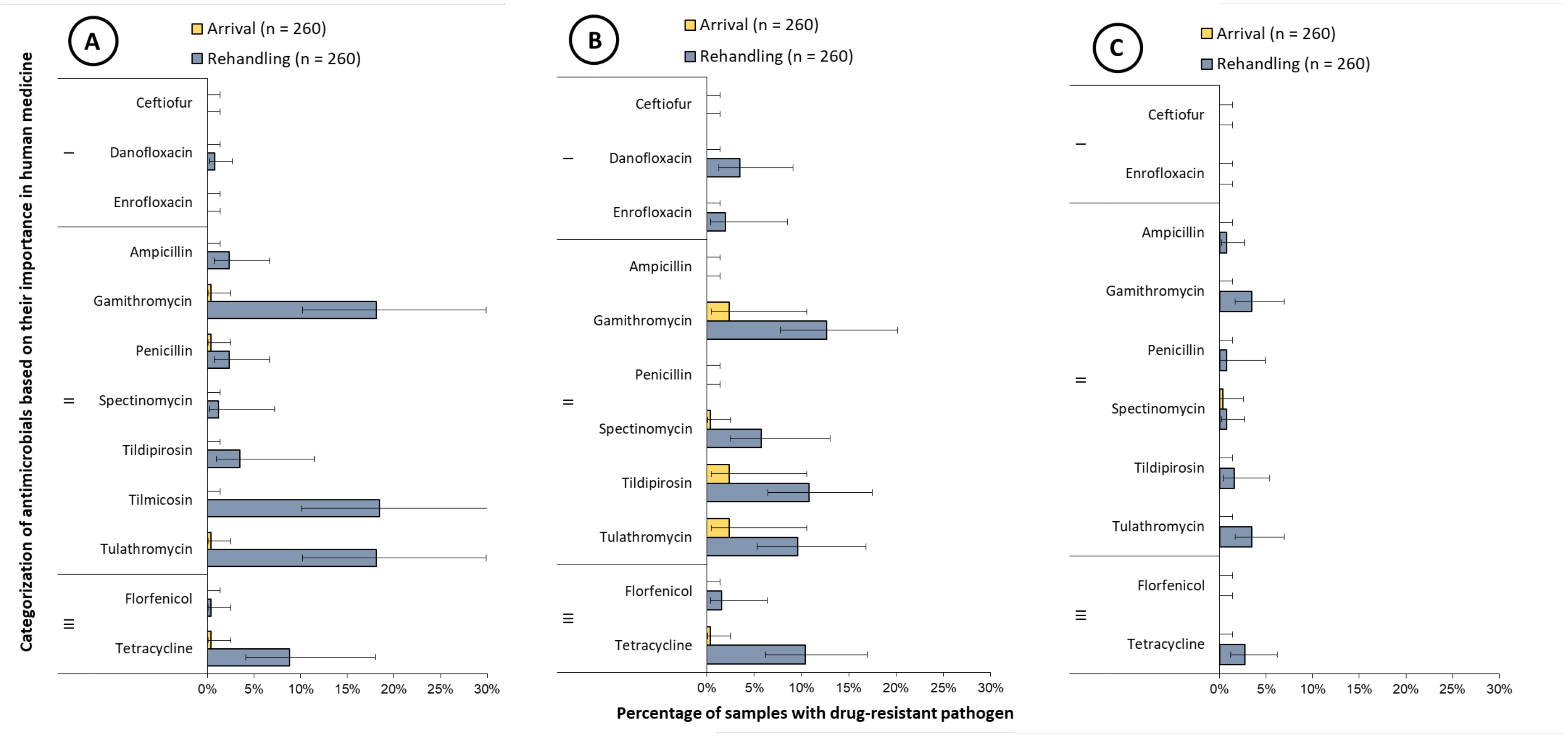

Supplementary Figure S2.14. Percentage of samples from yearlings with resistance to single antimicrobials in A) *Mannheimia haemolytica,* B) *Pasteurella multocida,* and C) *Histophilus somni* at feedlot arrival and at rehandling (*ASSETS* samples from 2022), adjusted for clustering by feedlot. Note that the maximum boundary for the x-axis is 30% in these figures.

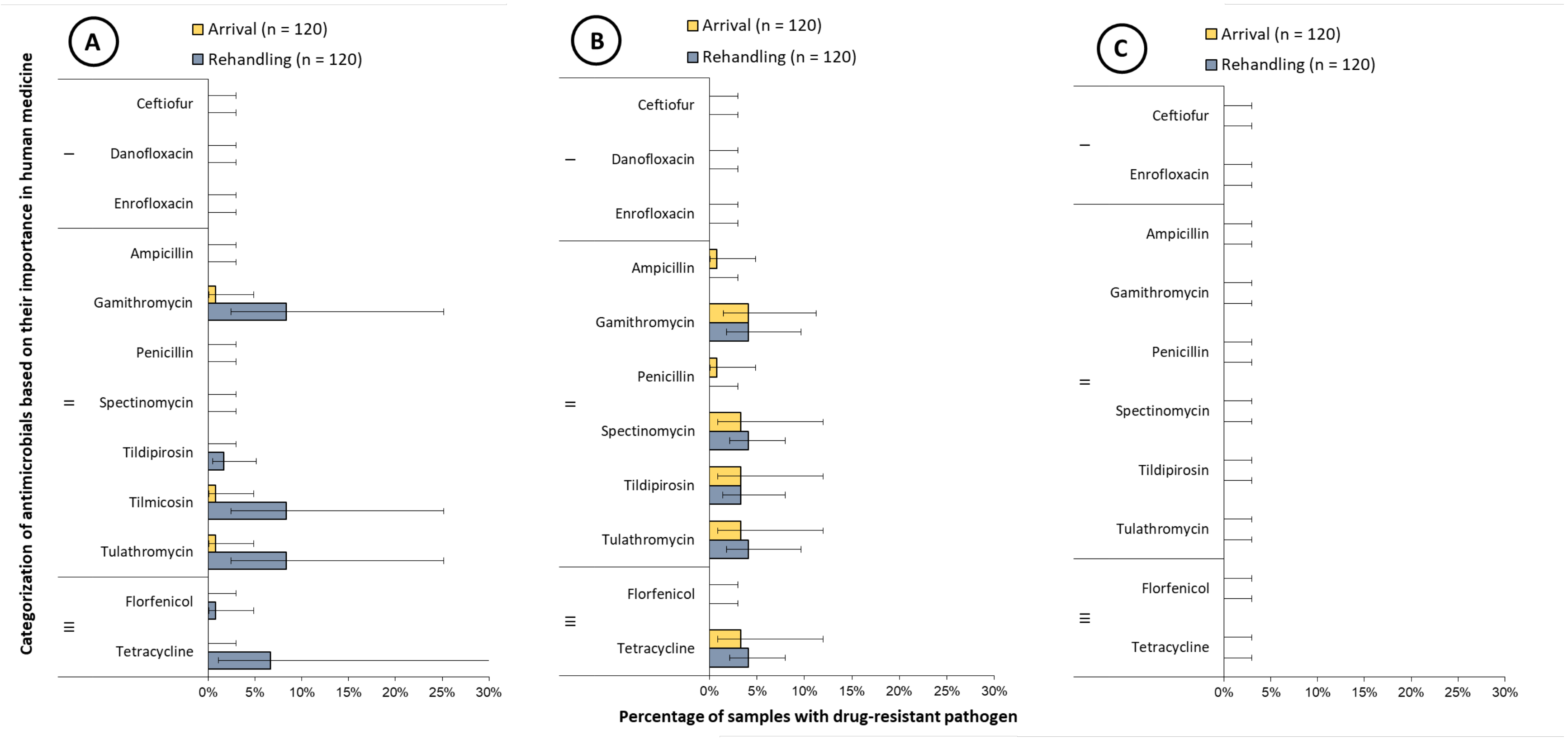

Supplement: Supplementary file 2 [file Data_Sheet_2.pdf]
