# Supplementary material for: Laboratory tests for bovine respiratory bacteria and antimicrobial resistance in commercial feedlot cattle: comparing culture, long-read metagenomics, and recombinase polymerase amplification
Source: Front Microbiol. 2026 May 20;17:1806062. doi: 10.3389/fmicb.2026.1806062 (PMC13229862; doi:10.3389/fmicb.2026.1806062)
Supplement: Supplementary file 4 [file Data_Sheet_4.pdf]

Supplement Material 4: Supporting information. Summary of detected organisms by test.

Supplementary Table S4.1. Summary of combinations of detected organisms (*Mannheimia haemolytica, Pasteurella multocida, Histophilus somni, Mycoplasmopsis bovis*) by each test across all study samples (n=760 samples).

Combinations of detected organisms	Percentage of samples (count)[1]		
	Culture or qPCR[2]	Metagenomics[3]	RPA
M. haemolytica alone	13.2% (100)	12.0% (91)	5.7% (43)
P. multocida alone	13.7% (104)	9.1% (69)	5.7% (43)
H. somni alone	2.6% (20)	2.4% (18)	3.3% (25)
M. bovis alone	11.2% (85)	5.8% (44)	14.2% (108)
M. haemolytica and *P. multocida*	3.7% (28)	4.1% (31)	2.4% (18)
P. multocida and *H. somni*	0.8% (6)	0.8% (6)	0.5% (4)
H. somni and *M. bovis*	0.8% (6)	0.7% (5)	1.7% (13)
M. haemolytica and *H. somni*	0.1% (1)	3.4% (26)	0.4% (3)
M. haemolytica and *M. bovis*	5.1% (39)	0.5% (4)	1.4% (11)
P. multocida and *M. bovis*	3.7% (28)	0.7% (5)	0.4% (3)
M. haemolytica, P. multocida, and *H. somni*	0.1% (1)	2.9% (22)	0.3% (2)
P. multocida, H. somni, and *M. bovis*	0.3% (2)	0.1% (1)	0.5% (4)
M. haemolytica, H. somni, and *M. bovis*	0.8% (6)	0.9% (7)	0.3% (2)
M. haemolytica, P. multocida, and *M. bovis*	1.1% (8)	0.1% (1)	0.3% (2)
M. haemolytica, P. multocida, H. somni, and *M. bovis*	0.0% (0)	1.3% (10)	1.3% (10)
None of the above organisms detected	42.9% (326)	55.3% (420)	61.7% (469)

RPA – recombinase polymerase amplification

[1] 760 samples collected from 19 feedlots, 20 samples per pen, and 1 pen per feedlot, at arrival processing and 14 days on feed (13 pens of fall-placed calves, 6 pens of yearlings).
[2] *M. haemolytica*, *P. multocida*, and *H. somni* detected with bacterial culture as described. *M. bovis* detected with qPCR.
[3] Metagenomics result classified as positive based on theoretical coverage of >0.29X for *M. haemolytica*, >0.73X for *P. multocida*, and >0.02X for *H. somni*. A cutoff of ≥2 reads was required to obtain a specificity ≥ 0.90 for metagenomic detection of *M. bovis*.
