# Supplementary material for: Laboratory tests for bovine respiratory bacteria and antimicrobial resistance in commercial feedlot cattle: comparing culture, long-read metagenomics, and recombinase polymerase amplification
Source: Front Microbiol. 2026 May 20;17:1806062. doi: 10.3389/fmicb.2026.1806062 (PMC13229862; doi:10.3389/fmicb.2026.1806062)
Supplement: Supplementary file 5 [file Data_Sheet_5.pdf]

Supplementary Material 5: Positive and negative predictive value results tables.

Supplementary Table S5.1. Positive (PPV) and negative predictive values (NPV) for detection of *M. haemolytica, P. multocida,* or *H. somni* with resistance to gamithromycin (GAM), tildipirosin (TILD), tilmicosin (TILM), and/or tulathromycin (TULA) from culture and antimicrobial susceptibility testing (AST), *msrE-mphE*[1], *erm(42),* and/or *estT* from long-read metagenomics, or *msrE-mphE*[1] and/or *erm(42)* from recombinase polymerase amplification (RPA) estimated based on sensitivity and specificity from Bayesian latent class models.

**Inputs**	**GAM, TILD, TILM, and/or TULA**		***msrE-mphE*[1], *erm(42)*, and/or *estT***		***msrE-mphE*[1] and/or *erm(42)***	
**Test**	**AST**		**Metagenomics**		**RPA**	
**Sensitivity[2]**	**0.61**		**0.24**		**0.23**	
**Specificity[2]**	**0.98**		**0.98**		**0.79**	
**Prior probability of infection**	**PPV**	**NPV**	**PPV**	**NPV**	**PPV**	**NPV**
**0**	0	1	0	1	0	1
**0.01**	0.24	1	0.11	0.99	0.01	0.99
**0.05**	0.62	0.98	0.39	0.96	0.05	0.95
**0.10**	0.77	0.96	0.57	0.92	0.11	0.90
**0.15**	0.84	0.93	0.68	0.88	0.16	0.85
**0.20**	0.88	0.91	0.75	0.84	0.22	0.80
**0.25**	0.91	0.88	0.80	0.79	0.27	0.75
**0.30**	0.93	0.85	0.84	0.75	0.32	0.71
**0.40**	0.95	0.79	0.89	0.66	0.42	0.61
**0.50**	0.97	0.72	0.92	0.56	0.52	0.51
**0.60**	0.98	0.63	0.95	0.46	0.62	0.41
**0.70**	0.99	0.52	0.97	0.36	0.72	0.31
**0.75**	0.99	0.46	0.97	0.30	0.77	0.25
**0.80**	0.99	0.39	0.98	0.24	0.81	0.20
**0.85**	0.99	0.31	0.99	0.19	0.86	0.15
**0.90**	1	0.22	0.99	0.13	0.91	0.10
**0.95**	1	0.12	1	0.06	0.95	0.05
**0.99**	1	0.02	1	0.01	0.99	0.01
**1**	1	0	1	0	1	0

Predictive values >75% shaded; values >75% consistent across two (AST and metagenomics) or three tests were shaded more darkly.

[1] *msrE-mphE*: includes any samples positive for *msrE* or *mphE*. [2] Sensitivity and Specificity from Table 9.

Supplementary Table S5.2. Positive (PPV) and negative predictive values (NPV) for detection of *M. haemolytica, P. multocida,* or *H. somni* with resistance to tetracycline from culture and antimicrobial susceptibility testing (AST), *tet(H)* from long-read metagenomics, or *tnpA-tet(H)* and/or *ebrB-tet(H)* from recombinase polymerase amplification (RPA) estimated based on sensitivity and specificity from Bayesian latent class models.

**Inputs**	**Tetracycline**		***Tet(H)***		***tnpA-tet(H)* and/or *ebrB-tet(H)***	
**Test**	**AST**		**Metagenomics**		**RPA**	
**Sensitivity**[1]	**0.46**		**0.43**		**0.20**	
**Specificity**[1]	**0.99**		**0.94**		**0.91**	
**Prior probability of infection**	**PPV**	**NPV**	**PPV**	**NPV**	**PPV**	**NPV**
**0**	0	1	0	1	0	1
**0.01**	0.32	0.99	0.07	0.99	0.02	0.99
**0.05**	0.71	0.97	0.27	0.97	0.10	0.96
**0.10**	0.84	0.94	0.44	0.94	0.20	0.91
**0.15**	0.89	0.91	0.56	0.9	0.28	0.87
**0.20**	0.92	0.88	0.64	0.87	0.36	0.82
**0.25**	0.94	0.85	0.7	0.83	0.43	0.77
**0.30**	0.95	0.81	0.75	0.79	0.49	0.77
**0.40**	0.97	0.73	0.83	0.71	0.60	0.63
**0.50**	0.98	0.65	0.88	0.62	0.69	0.53
**0.60**	0.99	0.55	0.91	0.52	0.77	0.43
**0.70**	0.99	0.44	0.94	0.41	0.84	0.33
**0.75**	0.99	0.38	0.96	0.35	0.87	0.27
**0.80**	0.99	0.31	0.97	0.29	0.90	0.22
**0.85**	1	0.24	0.98	0.23	0.93	0.17
**0.90**	1	0.17	0.98	0.15	0.953	0.11
**0.95**	1	0.09	0.99	0.08	0.98	0.06
**0.99**	1	0.02	1	0.02	1	0.01
**1**	1	0	1	0	1	0

Predictive values >75% shaded; values >75% consistent across two (AST and metagenomics) or three tests were shaded more darkly.
[1]Sensitivity and Specificity from Table 9.
